# Pathological Anatomy

**Published:** 1837-04-01

**Authors:** 


### Medico-Chirurgical Review,

### No. LII.

[No. 12 of a Decennial Series.]

JANUARY 1, TO APRIL 1, 1837.

### PATHOLOGICAL ANATOMY.

I. OUTLINES OF HUMAN PATHOLOGY. By *Herbert Mayo*, F.R.S., &c. &c. &c. Parts 1 and 2, 8vo. stitched, pp. 264—595. London, 1835-6.

II. LECTURES ON THE NERVOUS SYSTEM, AND ITS DISEASES. By *Marshall Hall*, M.D. F.R.S., &c. 8vo. pp. 171. London, 1836.

III. THE HUMAN BRAIN, ITS CONFIGURATION, STRUCTURE, DEVELOPMENT, AND PHYSIOLOGY; ILLUSTRATED BY REFERENCES TO THE NERVOUS SYSTEM IN THE LOWER ORDERS OF ANIMALS. By *Samuel Solly*, Lecturer on Anatomy and Physiology in St. Thomas's Hospital. 12mo. pp. 492—XII. Plates, London, 1836.

IV. LECTURES ON THE MORBID ANATOMY OF THE SEROUS AND MUCOUS MEMBRANES. In two Volumes. Volume 1. ON THE SEROUS MEMBRANES. By *Thomas Hodgkin*, M.D., &c. &c. 8vo. pp. 402. London, 1836.

V. OUTLINES OF PHYSIOLOGY. By *Herbert Mayo*, F.R.S., &c. &c. Fourth Edition. 8vo. pp. 534, London, 1837.

VI. PATHOLOGICAL AND SURGICAL OBSERVATIONS RELATING TO INJURIES OF THE SPINAL CORD. By Sir *Benjamin Brodie*, Bart., Serjeant Surgeon to the King, and Surgeon to St. George's Hospital. (Med. Chir. Transactions, Vol. XX.)

VII. ILLUSTRATIONS OF THE ELEMENTARY FORMS OF DISEASE. By *Robert Carswell*, M.D., &c. Fasciculus Eleventh. Analogous Tissues.

WE observed in the last number of this Journal, that we proposed to continue and extend our plan, of devoting at least one special article each quarter to the important subject of pathological anatomy. We say special article, for, probably there is scarcely *any* article to be found in the pages of a Medical Periodical, which has not more or less of such a pathological character.

A glance at the works whose titles we have here transcribed, or, indeed, at those titles only, must convince both the young student and experienced practitioner of the necessity of keeping pace with the advancing information of the day. Men of high talent are incessantly occupied in widening the boundaries of pathological knowledge, and a few years effect a very sensible difference in its limits.

The necessity for constant reading and study, does not simply depend on the daily accumulation of fresh facts and observations, but on the generalizations which are founded on the old. It is so short a time, since the field of Morbid Anatomy was first broken, that, although it has been ploughed, the crop is not yet ripe; the sickle is to come, and the sheaves are to be bound; nay, there are parts, over which neither plough nor harrow has yet travelled.

Periodical Reviews of the existing state of knowledge in a progressive science like medicine, are of equal interest and utility. Such reviews are peculiarly adapted for a journal with long intervals between its periods of publication. To a certain extent, almost every analytical article may be said to be of this description, for each work, at least each work of any value, is a mirror, in which the actual state of knowledge is reflected. But a more elaborate and comprehensive digest is desirable, and the reviewer who should regularly offer one, would confer no slight benefit upon his readers. The labour of such a task precludes the probability of its performance, for it would be totally incommensurate with any prize which even successful criticism has to give. We do not affect to undertake it, but shall content ourselves with occasionally presenting an article, which, founded upon several works, will gauge, in some measure, the scientific condition of the period.

### I. PHYSIOLOGY OF THE SPINAL CORD.

In the 49th Number of this Journal, we took up the subject of the pathology of the cerebrum, and promised, at an early opportunity, to proceed to that of the spinal cord. This we shall now do. We shall take the works of Dr. Marshall Hall, of Mr. Mayo, and of Sir Benjamin Brodie, especially of the latter, for the text.

In order that the morbid affections of the cord may be clearly understood, it is necessary to say a few words on its anatomy and functions. The cord may be considered as terminating above, at the decussation of the anterior pyramids, or commencement of the medulla oblongata—and as ending below, opposite to the first or second lumbar vertebra, where it branches into the "cauda equina."

The cord represents a column, with a deeper anterior median furrow, and a shallower posterior one. There are also on each side two lateral grooves, if grooves they deserve to be called, from the anterior of which issue the anterior roots of the spinal nerves, and from the posterior of which spring their posterior roots.

The cord is composed of white nervous matter and of grey. The latter, the "cineritious neurine" is included within the white, which is external. The grey matter may be broadly said to represent, in each lateral half of the cord, a small segment of a circle, the concavity of which is turned outwards —the anterior horn forwards—the posterior backwards towards the origin of the posterior roots of the nerves—and the convexity is turned inwards, or towards the mesial line, across which runs a connecting band of grey matter, forming the union or commissure between the portions in the two halves of the cord. The quantity of grey matter is greatest at the lower part of the neck, and about the lowest dorsal and upper lumbar vertebræ; in fact, where the largest nerves spring from the cord. Each lateral half of the cord is divided into two columns. All before the posterior horn of grey matter is the antero-lateral column—all behind it is the posterior column, the former considerably larger than the latter. At the bottom of the anterior furrow are transverse fibres, constituting a commissure between the lateral segments. The grey matter of the cord is vascular and pulpy—the white is distinctly fibrous.

The exact origins of the spinal nerves, particularly of their posterior roots, are not determined—indeed there is some difference of opinion respecting the number of columns in the cord, and their divisions.

The anterior portion of the cord is continuous with the anterior pyramids, and olivary bodies of the medulla oblongata. The posterior portion of the cord is continuous with the posterior pyramids, or restiform bodies. The former may be loosely said to be expanded into the cerebral hemispheres—the latter into the cerebellum. The dissections of Rolando and of Mr. Solly have shewn that the anterior pyramids are also connected with the cerebellum.

It is well known that the anterior roots of the spinal nerves, devoid of any ganglion on them, constitute their motor part—while the posterior roots, which have a ganglion, form their sensitive portion. The anterior portion of the cord has, therefore, motor powers, and the posterior portion of the cord has sensitive. The precise line of demarcation between the columns for motion and sensation, has not been ascertained.

Looking at the spinal cord and nerves as engaged only in the function of sensation and of voluntary motion, the motor fibres of nervous matter may be said to arise in the encephalon, to pass down and compose the anterior column of the cord, to issue from it under the form of the anterior roots of the nerve, and then, constituting *its* motor portion, to be finally distributed in the muscular tissue. The sensitive fibres, on the contrary, take their rise on the surface or in the texture where sensation is, pass back to the cord, before entering which they present a ganglion, and on entering which they constitute the posterior roots of the nerve, and finally, in the posterior portion of the cord, they proceed to their destination, the encephalon. The circle is complete. The encephalon receives sensation from the surface through the sensitive fibres of the nerve, and it transmits the mandate and the power of contraction to the mobile organ by the motor fibres.

This arrangement is one for voluntary motion, and for voluntary motion only. The power is exclusively situated in the encephalon—the cord, like the nerves is a mere medium of transmission. There are reasons for believing that the cord has a degree of independent power, and for supposing that that power is the result of the combination of grey and of white matter in its structure. In order to bring this subject before our readers, we will introduce Mr. Mayo.

"The dependence of the spinal cord upon the enkephalon is not exactly of the same nature with the dependence of the nerves upon the cord. The spinal nerves arise—that is, begin or end—in the spinal marrow, and have no power independently of their connexion and continuity with it. The spinal cord in the same way is a production of the enkephalon, and has no part in consciousness but when in continuity with it: still it possesses forces which are not derived from the enkephalon. The spinal cord with the spinal nerves forms thirty-one separate organs; each organ consisting of a segment of the grey and white matter of the spinal cord, and the pair of nerves originating in it. Each of these organs in one sense is independent of all the rest; one of the sentient nerves belonging to it being irritated, the impression which it conveys to the segment of the cord in which it rises is capable of exciting there an impulse to motion, which is transmitted by the voluntary nerve to the muscles. Each of these thirty-one organs is to its corresponding segment of the body, the organ of sensation, volition, and the commonest instinct. It is true that the white fibres which communicate with the enkephalon, carry upwards the impressions of sensation, and bring downwards the cerebral impulses either of deliberate design or passion. But the organs below are already perfect to the extent assigned to them, although incapable of ministering to consciousness, unless when continuous with the enkephalon. Accordingly, in some cases of privation of sense of motion in the legs through disease affecting the middle of the spinal cord, I have seen so much independent power remain in them, that pricking or tickling the foot, which yet excited no sensation, and was unknown to the patient, was nevertheless followed by its retraction." 154.

Facts of this description have been evolved by Dr. Marshall Hall into an elaborate and ingenious theory—that of what he terms the reflex function of the spinal cord and the medulla oblongata. On a previous occasion, we alluded to Dr. Hall's hypothesis—on the present, we may present a short, but accurate sketch of it.

After enumerating the nerves of sense and voluntary motion, which own the cerebrum as their centre, he proceeds :—

" A peculiar set of nerves constitute, with the true spinal marrow as their *axis,* the second subdivision of the nervous system. As those of the former subdivision were distinguished into sentient and voluntary, these may be distinguished into the *excitor* and *motory.* The first, or the excitor nerves, pursue their course principally from *internal* surfaces, characterized by peculiar excitabilities, *to* the true medulla oblongata and spinalis; the second, or the motor nerves, pursue a reflex course *from* that medulla to muscles having peculiar actions concerned principally in ingestion and egestion. The motions connected with the former, or cerebral subdivision, are sometimes, nay, frequently, *spontaneous:* those connected with the true spinal are, I believe, *always excited.*" 14.

In the true medulla, the centre of *this* system, he includes the tubercula quadrigemina and the medulla oblongata. The excitor nerves he enumerates, as the fifth, the pneumogastric, and the posterior spinal nerves—the reflex or motor nerves connected with those excitors, he pronounces to be the fourth—the sixth—the seventh (the portio dura)—the pneumogastric—the spinal accessory—the phrenic—the inferior external respiratory (of Bell) —the spinal nerves.

The physiological doctrine founded on the preceding anatomical classification, or rather on which the classification depends, is introduced by the details of two experiments.

1. A horse was struck with the poll-axe over the anterior lobes of the brain. It fell instantly, as if struck with a thunderbolt; it was convulsed, and then remained motionless. It shortly began to breathe, and continued to breathe freely by the diaphragm.

When lacerated or pricked by a sharp or pointed instrument, as a *pin* or a *nail,* on any part of the face or surface of the body, it was totally motionless, manifesting no evidence of sensation or volition.

When, on the other hand, the *eyelash* was touched with a *straw*, the eye-lid was forcibly closed by the action of the *orbicularis*. When the cornea was touched, the eye-ball revolved outward by the action of the *abducens*. When the verge of the anus was touched, the *sphincter* contracted forcibly, the tail was raised, the vulva was drawn towards the anus.

The upper part of the medulla oblongata was now destroyed by an instrument passed through the orifice made by the poll-axe: there were violent convulsions; the respiration ceased, and the eye-lid and eye-ball remained motionless on the application of stimuli.

2. The second experiment we may suppose to be performed by the lecturer before his class. A frog, not a horse, is the subject of it.

"You observe, says Dr. Hall this living frog: its sentient and voluntary functions are obvious. I divide the spinal marrow, below the occiput, with these scissors: all is still. There is not a trace of *spontaneous* motion. The animal would remain in this very form and position, without change, until *all* signs of vitality were extinct. But now I pinch a toe with the forceps. You see how both posterior extremities are moved. All is now still again; there is no spontaneous motion, no *sign of pain* from the wound made in the neck. It is without sensibility—without volition; the *power* to move remains—the *will* is extinct. I now pinch the integument. You observe the result—the immediate recurrence of excitomotory phenomena.

I now destroy the whole spinal marrow with this probe. It is in vain that I pinch the toes; the animal, the limbs are motionless!

Could the former *excited* motions be those of irritability? I will try the truth of this suggestion by seeing whether, now that the axis of the excito-motory system is destroyed, with its phenomena, the application of a slight galvanic shock will prove the subsistence of irritability. You see how instantaneously and forcibly the muscles are stimulated to contraction." 19.

Dr. Hall observes that the distinction between the motions of the "excito-motory system" and those of volition, on the one hand, and between them and those of irritability on the other, are perfectly distinct. The former conclusion may be readily admitted—the latter is surrounded with more obscurity and difficulty. Of course, supposing the experiment quite accurate, it is clear that what is denominated irritability remains after the medulla is destroyed; but the exact distinctions between irritability, and what we may christen "excito-motility," are perhaps uncertain. But to proceed.

Dr. Hall remarks that perception, a mental phenomenon, results from impressions derived from the senses—that volition is a subsequent mental act—that voluntary motion may ensue—and thus that the motions which flow from sensation always imply volition. Now it appears to us that exception may be taken to this unconditional proposition. The contractility of the iris is, if not dependent on, at least associated with, the sensibility of the retina. Dr. Hall does not admit either the optic nerve or the third nerve into his excito-motory system. The adaptation of the eye to near and to remote objects is not, apparently, voluntary, yet is probably dependent on muscular action. We must suppose the action of the muscles of the internal ear, to be regulated by impressions made on the acoustic nerve, yet, so far as we know, their action is not voluntary. To speak strictly, then, we do not think it proved that "the motions which result from sensation *always* imply volition."

3. The motions which belong to the excito-motory system, are never spontaneous, but always excited, the result of the application of an appropriate stimulus. The influence of that stimulus is carried along an excitor and incident nerve to that medulla oblongata, or medulla spinalis, and reflected thence along other reflex or motor nerves. Thus the incident excitor nerves, the medulla, and the reflex motor nerves, constitute the system.

"They remain, as I have already stated, after the centre of the cerebral system has been removed by experiment, or destroyed by disease. Their distribution takes place principally about the larynx and pharynx, in connexion with the medulla oblongata; and about the sphincters, in connexion with the lower part of the spinal marrow; and hence they especially guard the *orifices* and *exits* of the animal frame. Other parts of the system govern the acts of *ingestion*,—deglutition, and respiration;—and the acts of *excretion*,—of the fæces, urine, and semen. A third portion of the system gives general tone to the muscular egestion, and consequently to the limbs." 21.

4. Proceeding to details, Dr. Hall observes:—

"Filaments of the fifth pair of nerves are the excitors distributed upon the border of the eye-lid and surface of the eye-ball,—upon the nostrils,—probably upon the fauces,—certainly upon the face,—and are the *first* agents in inducing closure of the eye-lid, sneezing, vomiting, and sobbing, when the eye-lash is touched, the nostrils stimulated, the fauces irritated, or cold water is dashed upon the face. Other, motor, nerves convey the reflex influence from the medulla oblongata to the orbicularis, and the various respiratory muscles whose actions are combined to the acts of sneezing, vomiting, and sobbing.

Filaments of the pneumo-gastric are the excitors, when carbonic acid gas, or a drop of water comes into contact with the larynx,—when the dust of ipecacuanha is inhaled into the bronchia with the effect of inducing asthma,—in deglutition,—in ordinary respiration,—and in the act of vomiting produced by antimony in the stomach, and calculi in the gall-duct or ureter." 23.

Dr. Hall mentions one or two circumstances, not unworthy of attention. If, for example, the fifth nerve in the fauces be irritated, vomiting is induced; if, on the contrary, the eighth in the pharynx be excited, the act of swallowing follows. It has happened, that when a patient has wished to excite vomiting by tickling the *fauces* with a feather, he has, by passing it into the *pharynx*, induced such an action of the muscles of deglutition, as has drawn it into the œsophagus. A similar event has occurred with respect to the female catheter. Certain nerves being excited on introducing this instrument, an action of the muscles has been induced, which has drawn the catheter out of the fingers of the surgeon into the bladder.

5. Dr. Hall applies his theory to the function of respiration.

"In reference to respiration, several facts render it probable, although distinct experiments are required to render it certain, that the act of ordinary inspiration is *excited* by the contact of a certain proportion of carbonic acid with the filaments of the pneumo-gastric nerve in the lungs: Dr. Faraday particularly mentions the fact, that the respiration can be suspended longer after repeated deep inspirations, by which the air of the lungs is completely renewed, than in ordinary circumstances; in Hook's celebrated experiment, a stream of atmospheric air was driven through the trachea, the lungs, and incisions made through the pleura in a living dog: the animal made no effort to inspire whilst this stream was continuous, but, when it was interrupted, the efforts of inspiration were violent and convulsive. In this manner we may understand the *impossibility* of suspending the respiration beyond a certain period. In this manner we understand why an animal dies convulsed from submersion under water if the pneumo-gastric nerves be entire, but without convulsions if they be divided." 24.

Legallois, M. Flourens, Sir Charles Bell, have considered the acts of respiration as spontaneous, and the medulla oblongata as their source and primum mobile. Dr. Hall makes respiration but a part of his excito-motory system, and the excitory nerves he enumerates as principally filaments of the fifth, the pneumo-gastric, and the spinal nerves. The pneumo-gastric combines the action of the different muscles together, into acts of respiration, &c. in addition to its being one of the excitors of these actions.

The respiration, like all the functions of the excito-motory class, is a mixed function, that is, may be voluntary or excited. When both the pneumo-gastric nerves have been divided it is cerebral—when the cerebrum is removed, it is excito-motory—when both the cerebral and excito-motory influences have been subtracted, it is annihilated.

6. Deglutition is an excited act. The presence of some substance to be swallowed, some stimulus, excites it. It also may be voluntary, but it is curious, that it is impossible to perform the act of swallowing three or four times in rapid succession without taking something into the mouth. Dr. Hall explains this by saying that the absence of an excitant annuls the act, which, if it take place at all, must be an excited act. To us, however, it appears that the explanation must be rather sought in the physical construction of the pharynx, and in the fatigue of attempting to contract a tube several times in quick succession, without giving it any thing to contract on. We do not experience the same difficulty in other parts of the "excito-motory system," for instance, in the endeavour to wink very often and with great rapidity. As deglutition is the associated action of many muscles, it must of course be proportionately difficult.

"I must now mention another fact. The whole *tone* of the muscular system is the result of an excito-motory function. The limbs of an animal, or of a part of an animal separated from the influence of the cerebrum, become relaxed on destroying the spinal marrow. If we remove the tail and the rectum from a recently decapitated turtle, the sphincter retains its circular form, the tail its firmness,—phenomena which cease entirely on withdrawing the portion of spinal marrow remaining within the caudal spinal canal." 26.

Dr. Hall here relates some experiments upon the turtle, detailed in a paper published in the Transactions of the Royal Society, for 1833.

A turtle was decapitated by means of a knife, which divided the second or third vertebra.

"The head being placed upon the table for observation, it was first remarked that the mouth opened and shut, and that the submaxillary integuments descended and ascended, alternately, from time to time, replacing the acts of respiration. I touched the eye or eye-lid with a probe. It was immediately closed: the other eye closed simultaneously. I then touched the nostril with a probe. The mouth was immediately opened widely, and the submaxillary membranes descended. This effect was especially induced on touching the nasal fringes situated just within the anterior part of the maxilla. I passed the probe up the trachea and touched the larynx. This was immediately followed by a forcible convulsive contraction of the muscles annexed to it. Having made and repeated these observations, I gently withdrew the medulla and brain. All the phenomena ceased from that moment. The eye, the nostrils, the larynx, were stimulated, but no movement followed.

The next observations were made upon the other parts of the animal. The limbs, the tail, were stimulated by a pointed instrument or a lighted taper. They were immediately moved with rapidity. The sphincter was perfectly circular and closed; it was contracted still more forcibly on the application of a stimulus. The limbs and the tail possessed a certain degree of firmness or tone, recoiled on being drawn from their position, and moved with energy on the application of the stimulus. On withdrawing the spinal marrow gently out of its canal, all these phenomena ceased. The limbs were no longer obedient to stimuli, and became perfectly flaccid, having lost all their resilience. The sphincter lost its circular form and its contracted state, becoming lax, flaccid, and shapeless. The tail was flaccid, and unmoved on the application of stimuli." 27.

These phenomena subsist in distinct portions of the medulla. Thus, if the lower extremities and tail be separated together, they will, when stimulated, answer to those stimuli in the manner mentioned. Dr. Hall has found that similar movements of the limbs are excited by pinching the lateral nerves, as they leave the spine, continuously, by the forceps. The influence of this stimulus is not only *reflected* upon the limbs, but it is *retrograde* in its course, passing from a nerve proceeding from the middle part of the spine, *forwards* to the anterior, as well as backwards to the posterior extremities. This experiment seems to Dr. Hall to present us with the simplest type of some spasmodic diseases, especially of the traumatic tetanus. From other experiments upon the frog, he has ascertained that the extreme filaments of the excitor nerves are more impressible by stimuli, than the same nerves in their course. This is what we might anticipate.

Leaving the excito-motory system, Dr. Hall advances to the third division of the nervous system, the ganglionic. The novelty in his notice of this system attaches itself to his suggestion respecting the office of the ganglia on the fifth, and on the posterior roots of the spinal nerves. Why have those nerves ganglia? It is true that they have been proved to be endowed with sensation. But it does not therefore follow that sensation is the only endowment that they have, *if* sensation be the gift of the ganglion. The following is Dr. Hall's reply to the question, and we cannot conscientiously consider it a conclusive one.

" There is," says he, "an internal nerve* for formation, nutrition, secretion, &c. 2. This nerve is ganglionic. 3. There are external organs and structures requiring nutrition, &c. 4. There are also external ganglionic nerves. The inference is plain, that these constitute the external ganglionic sub-system. The fifth especially abounds with ganglia.

It is true that the semilunar and external spinal ganglia differ in appearance from the ganglia of the sympathetic, as Sir Charles Bell has well displayed. What is the nature of this difference? To this question I find no reply in authors. It is plain, however, that the difference consists in their being, alone, *plexic*. The internal ganglionic nerve is purely nutrient: its ganglia are simple. The external involve sentient, and I believe excitory, nerves, with the nutrient; they combine, therefore, the appearances of the plexus and of the ganglion.

* The sympathetic.

I must add another argument upon this point. If the sensation of the face be lost by paralysis, arising from disease of the *brain*, the eye is safe; but if the same event occur from compression or destruction of the *fifth*, *within* the cranium, by disease, or in an experiment, the eye ceases to be nourished, and becomes destroyed! In the former case the nerve of sensation merely has suffered; in the latter the nerve of nutrition, as well as sensation, has been involved in the disease or injury." 31.

Such is the theory of Dr. Marshall Hall—such the nature of that "reflex function of the spinal marrow and medulla oblongata," which he has introduced with much pains and much talent to the notice of the profession, and, we may add, of the scientific public.

It must be owned, that there are few objections even to its details; to its essential principles, none. It must be owned, that the facts which it embraces, it explains; and, while it explains, it gives them increased interest. If, then, it be true that all this is absolutely new to us—if the development of these ingenious views be the work of Dr. Hall, and of him only, he must be allowed to take his stand in the first rank of the physiologists of the day, a not contemptible distinction.

But these physiologists are by no means remarkable for brotherly love. The "abandonnement de soi," which has ever been the boast of abstract philosophy, is not usually a prominent trait in its votaries. The maitre de philosophie in the bourgeois gentilhomme, is the representative of the equanimity of his order, and infames, scelerats, coquins, are forms of argument, which do not seem to be inconsistent with the pursuit of truth.

In the last edition of Mr. Mayo's Physiology, we observe a note upon the Reflex Theory, which certainly is not calculated, and, perhaps, was not intended to be highly complimentary to Dr. Hall. Mr. Mayo vindicates as his own property, the spirit of the theory in question, and leaves to Dr. Hall the equivocal merit, of supporting *his* views by two poor experiments. It would be unjust to either gentleman not to give insertion to this note, and its brevity supports our sense of right. Here it is.

"Under the name of 'reflex function of the spinal cord,' Dr. M. Hall has investigated a principle, which was explicitly laid down in my Anatomical Commentaries, *published in* 1823 [Part II, page 138], in the following words:—

'An influence may be propagated from the sentient nerves of a part to their correspondent nerves of motion, *through the intervention of that part alone of the nervous centre to which they are mutually attached*. Thus in vertebral animals, in which alone the fact is questionable, when the spinal cord has been divided in two places, an injury of the skin of either region is followed by a distinct muscular action in that part. Again: if the brain is quickly removed from the head of a pigeon, leaving only the crura cerebri, together with the tubercles and the second and third nerves, on pinching the second nerve, the iris contracts.'

The same view and the same facts, carefully distinguished from the agency of sensation and volition, have been put forward in the former editions (as in the present) of my Outlines of Physiology.

The facts, which Dr. M. Hall has added to the knowledge existing on this subject long before he wrote, are, that after the spinal cord of a turtle has been divided, its sphincter ani continues to act; and that in a frog made tetanic by strychnine, division of the cord, in the back and in the neck, leaves each part tetanic.

These facts, valuable in themselves, are likewise good exemplifications of the principle above laid down. But their excellence, as examples, does not authorize

Dr. M. Hall *to affirm, in* 1833, that the independent power of the spinal cord 'had never been accurately distinguished from sensation and volition;' which he asserts in a paper in the Philosophical Transactions for that year, wherein he claims that distinction, and the principle it involves, as a discovery."

This note wears the livery of polemical philosophy, and is perhaps the commencement of that species of compliment, which experimenters on the nervous system liberally lavish on each other. But however that may be, it fortunately does not devolve on us to decide the respective merits of the parties, or to enter on a discussion of priorities and dates. Possibly *that* might strip both gentlemen of any great claim to originality, and shew that *some* of the facts had been established, previously to the publications of either. We have neither the wish nor the leisure to engage in disputes of this description, and shall take the respective merits of the parties pretty nearly at their own valuation.

But we must admit, in fairness to Dr. Hall, that granting Mr. Mayo all that he claims as exclusively his own, much praise is still due to the former gentleman. Mr. Mayo stated in an unimpressive manner, what was little more than a simple fact. Dr. Hall has evolved it into an extensive and ingenious theory, applied it to the solution of complex phenomena previously surrounded with obscurity, and has done a great deal towards clearing the way to a precise acquaintance with the functions of the nervous system. Such appears to us to be the meed which even a parsimonious critic must award to Dr. Hall. Mr. Mayo has done much towards the improvement of physiology, and he can afford to share his honours with his peers.

### II. INJURIES OF THE SPINAL CORD.

Our text is now the paper of Sir Benjamin Brodie, contained in the recently published volume of the Royal Medico-Chirurgical Society's Transactions. This paper is characterised, like all others, from the same source, by its conciseness, its inductive character, and the evidence it offers of being essentially founded on actual observation and experience.

Sir Benjamin remarks, that there is a lack of precise and positive information in pathological and surgical works, respecting the effects of injuries of the spine. The comparative difficulties of cadaveric examination have tended, no doubt, to occasion this. Our author presents his paper to the Society, with the view of contributing his share towards the removal of so serious a defect in surgical literature.

### *A.* WOUNDS OF THE SPINE.

On this part of the subject Sir Benjamin has nothing new to offer. Such wounds are usually fatal at an early period. He refers to Ollivier's Treatise on the Diseases of the Spinal Cord, for a notice of some experiments illustrating the effects of these injuries on animals, and for the histories of some cases, in which they occurred in man.

Mr. Mayo, in his Outlines of Pathology, quotes two instances of recovery after wounds of the spine.

*Case.* A young man was struck on the back of the neck with a poniard, three fingers' breadth below the left ear, the wound penetrating obliquely towards the right. Immediate loss of sense and motion of the whole body ensued, the head alone feeling. During the first few days, there was retention of urine and constipation; afterwards, involuntary discharge of urine. Towards the eighteenth day, feeling began to return on the left side; on the twentieth, the patient could move the digits of the left hand and foot; and feeling and motion of the left side increased daily. On the thirtieth day, feeling began to re-appear on the right side; shortly after, motion. In three months he was so recovered that he could walk a little; the left side, however, having kept in advance of the right.—*Morgagni.*

*Case* 2. A drummer of the National Guard of Paris was struck on the back of the neck with the point of a sabre, which was thrown at him: his limbs bent under him, and he fell. The left arm was palsied. This effect was permanent. The left leg was likewise affected with weakness: but this disappeared on the fourth day. On the thirteenth day it was accidentally discovered that the right side of the body, from the fourth rib downwards, had lost its feeling: this anæsthesia was permanent.—*Boyer.*

Before we proceed to the examination of other injuries of the spine, we may return for a moment to the functions of the spinal marrow. It is certain, in the first place, that it is an organ of transmission, and by distinct fasciculi, appropriated to sensation and volition, conveys an impression from its nerves to the brain, and the mandate of the will from the brain to the nerves. In the second place, each segment of the cord forms with its nerves a sort of circle in itself, sensitive fibrillæ going to it, motor fibrillæ going from it. The functions which result from this arrangement are the "reflex," and exist independently of communication with the encephalon. Finally, the cord must exercise an influence upon nutrition, although its amount is not well ascertained.

The following are the logical consequences deducible from the premises set down.

1. If those fibres, by which the cord conveys sensation to the encephalon are rendered, by disease or accident, unable to perform their functions, the parts of the body supplied by spinal nerves, whose origin is below the seat of lesion, will no longer *feel.* The affection is designated anæsthesia.

2. If those fibres which convey volition from the brain are alone affected, then voluntary motion only is lost.

3. If both sets of fibres are involved, sensation and motion are destroyed.

4. If only one side of the cord is affected, one side only of the body suffers. But if both sides of the cord are implicated, both sides of the body suffer.

5. Certain fibres of transmission in the cord may escape, and those only may be diseased, which are concerned in the "reflex function" of one or more segments. The arms may be palsied, while the lower limbs still feel and are capable of voluntary motion. In such a case it is clear that the continuity of the encephalon with that part of the cord giving off the nerves of the lower extremities still holds, while that of the encephalon with the portion of the cord giving rise to the nerves of the upper extremities does not.

### B. CONCUSSION OF THE SPINAL CORD.

We may now return to Sir Benjamin Brodie. He classes the injuries to which the spine is subject, in the following order.

*First.* Fractures of the vertebræ without displacement of the fractured surfaces.

*Secondly.* Fractures with depression or displacement of bone, diminishing the diameter of the spinal canal, and occasioning pressure on the spinal cord.

*Thirdly.* Fractures complicated with dislocation.

*Fourthly.* Dislocations not complicated with fracture.

*Fifthly.* Extravasations of blood on the surface of the membranes of the spinal cord. Such extravasations, however, rarely take place to any considerable extent, and bear no comparison to those which occur within the cavity of the cranium in consequence of a rupture of the substance of the brain, or a laceration of the middle meningeal artery.

*Sixthly.* A narrow clot of extravasated blood is sometimes discovered within the substance of the spinal cord. It is always of a very small size, but from its peculiar situation may be productive of the most dangerous symptoms.

*Seventhly.* Laceration of the spinal cord and its membranes. Of course it is more easy to conceive than to describe all the varieties of this kind of injury, which are met with in practice. The spinal cord may be separated through its whole substance; or it may be torn in one part and not in another. M. Ollivier describes a case in which the attachments of the nerves on one side were destroyed, while those on the other side were entire.

*Eighthly.* The minute organization of the spinal cord may suffer from a blow inflicted on the spine, even where there is neither fracture nor dislocation, and where the investing membranes do not appear to participate in any way in the effects of the injury.

This case is analogous to that of concussion of the brain, and it has accordingly been termed concussion of the spinal marrow. The ultimate texture of both organs is too subtle, to admit of distinct appreciation by our senses; and it cannot be a matter of surprise, if that texture should be susceptible of lesion from violence, incapable of appreciation. Whether, however, this explanation of the effects of concussion be correct or not, it is certain that the spinal marrow, like the brain, will, after severe shocks, exhibit alterations in its functions, or even absolute annihilation of them, independently of much *apparent* change of structure.

As Sir Benjamin observes, much worse consequences usually arise than from concussion of the brain; that, if the patient recovers, his recovery is more tedious: that if he dies, greater changes in the condition of the injured part are detected on dissection. But these differences in the effects of the injury are easily explained. The brain and its membranes completely occupy the cavity of the cranium, while the spinal cord and its membranes occupy only a part of the vertebral canal: and this being the case, it requires no great knowledge of mechanics to enable us to understand why the same degree of violence applied to the head, and spine, should occasion different degrees of mischief to the organs which they contain.

The symptoms of concussion of the spinal marrow exhibit a wide range. From the most trivial impairment of sensation or of motion, they extend to complete suspension of its functions, and to destruction of the individual. Mr. Mayo writes, that, in a case given by Boyer, complete paraplegia had been instantaneously produced by a fall into a ditch: the patient died. On dissection, no disease could be discovered either in the head or spinal canal. Frank mentions four fatal cases of concussion of the spine, on the post-mortem inspection of which no change in the spinal marrow was detected, or injury of the vertebral column.

Mr. Mayo relates the particulars of a case of a less fatal character. We give it as illustrative of the more ordinary consequences of concussion.

*Case.* A man, aged 50, was admitted between four and five years ago into the Middlesex Hospital, having fallen out of a loft into a stable, in such a manner as to pitch upon the juncture of his neck and back. He did not lose his senses, but on being lifted up, his arms and legs were found to be numb and powerless. In a few days he recovered the feeling and use of his legs; the numbness and weakness likewise gradually left the arms; but his hands remained affected, and continue so still. The hands are numb and weak; the thumbs and fingers are drawn inwards, and are incapable of complete extension. The treatment employed was cupping, blistering on the neck, and calomel. About six months after the accident, strychnine was used, but without advantage.

At an uncertain period after the accident, at least in those cases, which, proving fatal, admit of an examination, the central part of the cord, Sir Benjamin Brodie assures us, is found to be softer than natural, and its fibrous appearance is lost in that of a semi-fluid substance. If the patient survives for a longer period, the alteration of structure is perceptible in the whole diameter of the cord, and occupies from one to two inches, or even more, of its length; and at a still later period it has often proceeded so far as to determine in its complete dissolution. This softening is the common consequence of the injuries of the spine, and the ordinary cause of the dangerous symptoms which succeed them. It is regarded by some pathologists as the result of inflammatory action, but Sir Benjamin doubts, for the following reasons, the accuracy of that conclusion.

"1st. A minute examination of the injured part of the spinal cord will often enable us to detect the commencement of the softening process at a very early period, before sufficient time had elapsed for inflammation to become established, and before any symptoms of inflammation had shewn themselves. 2dly. The softened part of the cord, in the first instance, exhibits no appearance of increased vascularity. 3dly. Even where the softening process is so far advanced as to occasion complete disorganization of the spinal cord, the investing membranes, for the most part, exhibit their natural appearance, there being neither increased vascularity, nor the slightest effusion of lymph, or serum, or pus on their surface. 4thly. The symptoms, which mark the progress of these changes, are (as I shall shew hereafter) merely a combination of those which the concussion of the spinal cord has occasioned in the first instance, and which of course must have been wholly unconnected with inflammation." 125.

It is very true, that this disorganization never proceeds far, without enlargement of the small vessels, in other words, an inflammatory state ensuing. But Sir Benjamin regards this as the consequence of the lesion, not its cause, and he draws a comparison between the lesion itself and idiopathic ramollissement, which there are strong reasons for considering not essentially inflammatory.

The question is one of much difficulty as well as interest, and probably it is insusceptible of positive determination. The preceding arguments are strong, yet it may be urged in opposition to them: 1, *that*, in the worst, or in very severe cases of *mere* concussion, whether of the brain or spinal marrow, no lesion whatever has been found: 2, *that*, softening of the cord may be said to be consecutive to the injury, and although it is discoverable at an early period, yet low inflammatory action may be set up at a very early period too: 3, *that*, softening is a progressive alteration, tending to increase in intensity and in extent with length of time; such progressive lesions are more readily accounted for, by supposing them to depend on processes of an inflammatory character than on the contrary: 4, *that*, a violent injury to an important organ, adequately supplied with blood, is likely to give rise to inflammation of its structure: 5, *that*, softening of the spinal marrow is sometimes a sequela of decided inflammation. Take for example, the following case extracted by Mr. Mayo, from Ollivier's book. A gentleman, who was liable to epilepsy, complained of uneasiness of the throat, with difficulty of swallowing, accompanied by acute pain in the neck and occiput, followed by fever, embarrassed breathing, and vomiting; he then was seized with numbness of the left hand, which speedily extended up the arm: the right was immediately after affected in the same manner, and on the following day they were both paralytic. The legs were not in the least affected, nor the functions of the bladder or bowels. He died on the eighth day, having preserved his intellects to the last. *Inspection.*—There was extensive softening of the upper part of the cord, chiefly of the grey matter, which was of a rose colour, with a highly vascular state of the membranes. 6, *that*, softening after injury, is occasionally attended with unequivocal inflammation of the spinal membranes: 7, *that*, the treatment which usually succeeds best, is such as would be calculated to relieve inflammatory action, for example, local or cautious general depletion, counter-irritation, and mercury: 8, *that*, the argument drawn from the idiopathic softening of the brain is open to some degree of objection, as circumstances differ in many respects in the two affections; 9, and finally, *that* the absence of unnatural vascularity after death, would disprove, it is true, the prior existence of an active state of inflammation, but is not conclusive evidence of there having been none.

A candid consideration of what may be urged upon both sides of the question, will probably give rise to the conviction, that it is still open to accurate investigation. Our knowledge of the phenomena of inflammation of nervous matter is less exact, than it is in respect to many of the other tissues of the body. Experiments and injections might assist in elucidating points of this description, and indeed we may remark that less attention has been directed to what we may term pathological experiments than they deserve.

### C. SYMPTOMS DEPENDENT ON INJURIES OF THE CORD.

Sir Benjamin introduces the examination of these, by an attempt to classify them. The immediate symptoms, he says, admit of being referred, 1st, to concussion of the spinal cord; 2dly, to laceration or division of its substance; 3dly, to pressure made on it either by displacement of bone or extravasated blood. If inflammation of the membranes of the cord take place, other, and a secondary order of symptoms ensue. Taking a general view of them, they are observed to vary, 1st, according to the part of the spinal cord on which the injury has been inflicted: 2dly, according to the kind and degree of injury which the cord has sustained; 3dly, accordingly, as from accidental circumstances, that is from circumstances of which we cannot take cognizance, the life of the patient is prolonged for a shorter or longer period, or ultimately preserved.

Our author gives an account of the several symptoms in succession, and afterwards offers some general observations, with the view of connecting and completing their history.

1. *Paralysis of the Voluntary Muscles.* This is an obvious and intelligible effect of injury of the spinal cord. Its extent varies with the amount of that injury. If the spinal cord should be divided through its whole substance, or extensively lacerated, or subjected to any considerable degree of pressure, the paralysis is immediate and complete. If the injury be partial, certain muscles may be paralysed, while others retain their power of voluntary motion.

"Concussion of the spinal cord," continues our author, "often produces complete paralysis also; but more frequently the paralysis arising from it is partial. One limb may be paralytic and another not so; or in the same limb certain muscles may be thus affected, while others are still obedient to the will. In some cases the patient has the power of using his limbs while he is in the horizontal posture, yet he is unable to stand erect. Or the degree of paralysis may vary at different periods. Thus it may be complete at first; then, after three or four days, the power of motion may be in some degree restored; then it may be lost again. Sometimes, although the paralysis is complete, or nearly complete, in the first instance, so speedy a recovery takes place, that the patient is able to walk in the course of three or four weeks, or even sooner; or the converse of this may happen, so that, although there is no more than a weakness of the muscles immediately after the accident, complete paralysis may take place, sometimes gradually, at other times almost suddenly, after the lapse of several weeks." 130.

Paralysis of the lower limbs is more common than that of the upper. In some cases, in which the injury has affected the spinal cord in the lower part of the neck, the lower limbs are rendered paralytic, while there is either no paralysis, or a less degree of paralysis in the upper limbs. The explanation is not difficult, indeed it has been given in some preceding observations. But in caries of the spine the case is usually reversed. In *that* the upper limbs are frequently paralytic for some time before the lower. May not this depend on implication of the nerves of the upper limbs at their roots, or at their passage through the intervertebral foramina? The causes of paralysis in this complaint extend to the cord from without.

"A case is recorded by Mr. Stafford, in which there was paralysis of both the upper and the lower limbs, consequent on an injury of the loins, with fracture and displacement of the lumbar vertebræ; and another case fell under my own observation, in which paralysis of the upper limbs followed a contusion of the middle dorsal vertebræ. I conclude that such cases are no more than apparent exceptions to the general rule, of the paralysis being confined to the parts which are below the injured portion of the spinal cord. It is easy to suppose that the bones may be fractured or displaced in the loins or back, or that blood may be extravasated within the theca vertebralis in one or the other of these situations, while another part of the spinal cord, as high as the origin of the nerves which form the axillary plexus, suffers from the effects of concussion." 131.

Paralysis after an injury of the spine is always dangerous. Yet many persons recover. A gentleman was thrown from his horse, and received a severe blow on his back. Five weeks afterwards he became paralytic in his lower limbs; but at the expiration of fifteen or sixteen weeks more, he began to recover the use of them, and he was able to walk with the assistance of a stick, when Sir B. Brodie was consulted, about a year afterwards. If the paralysis is the result of the pressure of extravasated blood, it may be relieved by the absorption of the coagulum—if, of lesion of the cord, that lesion may be reparable.

Cases still more remarkable are on record. The cord is said to have been completely divided, without the occurrence of paralysis then or afterwards. In the present state of our knowledge on this subject, perhaps, a little incredulity is advisable.

2. *Muscular Spasms.* "A gentleman, in October 1827, was thrown from his horse, which afterwards rolled over him. His head was not struck, and the principal injury seemed to have fallen on the lower part of the spine. Soon after the fall, and while he was yet lying on the ground, his thighs were raised spontaneously towards the trunk, and this was followed by an involuntary tremulous motion of the lower limbs. Afterwards the lower limbs became paralytic, and continued in that state for a period of two months. At the end of that time he began to recover, and when I was consulted, about a year after the accident, he was able to walk without even the assistance of a stick.

In the observations on injuries of the brain which I formerly communicated to this Society,* I mentioned some circumstances which seem to show that the convulsions which occasionally occur at an early period after a severe blow on the head, are the consequence of a slight extravasation of blood, which is sufficient to operate as a cause of irritation, without actually destroying the functions of the brain. Whether the convulsions which took place in this instance had a similar origin, or arose from that disorganization of the spinal cord which is the most usual cause of paralysis after concussion, must be left to future observations to determine. The same symptoms sometimes occur at a later period. For example: a man, forty-five years of age, in January 1825, fell from a scaffold, and received a blow on his back. All the parts below the epigastrium became immediately paralysed. At the end of nine days it was observed, for the first time, that a slight involuntary action of the muscles of the thighs was induced when pressure was made accidentally on these parts. Afterwards severe cramps and painful convulsions took place, whenever pressure was made on any part of the body, or even by lifting up the bed-clothes. At last they were almost constant, so as continually to awaken him from his sleep. When he died, nine weeks after the accident, it was ascertained that there had been a fracture of the fourth dorsal vertebra, with such a degree of displacement as to produce a slight degree of pressure on the spinal cord. There was an abscess,

* Medico-Chirurgical Transactions, Vol. XIV. p. 370.

containing from four to six ounces of pus, communicating with the fracture, and extending into the posterior mediastinum. The membranes of the spinal cord and the spinal cord itself, presented a natural appearance externally, but on the latter being divided longitudinally, the central part of it was found to be in a softened state, so that on being macerated for a short time in water, it almost completely disappeared." 134.

Sir Benjamin relates another case, in which there were fracture and considerable displacement of the third and fourth lumbar vertebræ, attended with paralysis of the lower limbs. An attempt at reduction was attended with a partial degree of success. At the end of a month he became affected with slight involuntary motions of the lower limbs, and at the same time he began to recover the power of moving them voluntarily. At the expiration of four months from his admission he left the hospital, and Sir Benjamin has heard no more of him.

"In each of the two last-mentioned cases there was some degree of pressure on the spinal cord; and I am the more inclined to believe that this was the cause of the spasmodic affection of the muscles, as I have not met with any case in which it was proved by dissection, that this symptom existed in combination with disorganization of the cord, and independently of pressure on it." 155.

It appears to us that there are some reasons for imagining that organic alterations of the cord, may, as well as pressure, give rise to these affections of the muscles. Analogy seems to be in favour of the supposition. A short time ago we attended, with Mr. Skegg of St. Martin's Place, a young man who was suddenly attacked with apoplectic insensibility, hemiplegia on the right side, and severe spasms of the muscles of the left. At the end of thirty-six hours, or thereabouts, he died. On dissection, we found the left optic thalamus so softened that it formed a mere pulp. There was no other appreciable alteration of the brain.

Setting aside any analogical considerations, there appear to be facts connected with the diseases of the cord itself, which give a degree of probability to the supposition that spasms of the muscles may depend on disorganization of the medulla.

*Case.* In a case described by Dr. Abercrombie, the progress of the disease was slow, and lasted from October to the July following: the symptoms being coldness and numbness of the feet, with diminished power of motion: after several weeks, complete palsy of the legs, and inability to empty the bladder; tightness across the abdomen; spasms like opisthotonos. In the intermediate April, some amendment occurred, with the power of moving the legs when supported by crutches. Two days before death, the right arm became paralytic, and his speech impaired. The loss of sensation was never complete.

*Inspection.*—The whole cord was of a pale rose colour, and was in every part entirely diffluent. The medulla oblongata was softened on its anterior part, as well as the adjacent portion of the annular protuberance.

### *Affections of the Nerves of Sensation.*

If, says Sir Benjamin, the spinal cord be lacerated, or subjected to any considerable degree of pressure, the sensibility of the parts below the seat of the injury is totally destroyed. If the injury be in the situation of the sixth or seventh cervical vertebræ, the destruction of sensibility is frequently partial in the upper extremities, while it is complete in the trunk and lower extremities; but if it correspond with the two vertebræ immediately above these, the patient, during the short remaining period of his life, presents the extraordinary phenomenon of a living head, with its sensibility and muscular powers unimpaired, attached to a trunk and extremities of the existence of which he is totally unconscious.

In cases of concussion of the spinal cord, there are the same varieties with respect to the destruction of sensibility, as there are with respect to that of voluntary motion. It may be complete, incomplete, partially complete. Sometimes the skin appears insensible, while the patient is conscious of pressure made on the more deeply seated parts.

"Not unfrequently, unusual sensations are referred to parts, the nerves of which are actually incapable of conveying to the sensorium the impressions made by mechanical pressure, or the application of heat. Sometimes the patient complains that he feels as if he had been everywhere severely bruised; or he has a sense of burning, or of tightness and constriction. In many instances, the destruction of sensibility is incomplete at first, but becomes complete afterwards, as the process of softening makes progress in the injured portion of the spinal cord. Where recovery takes place, the restoration of sensibility usually precedes that of the power of voluntary motion; so that the patient may be quite sensible of external impressions, while he is still incapable of employing his muscles for any useful purpose. The last observations apply equally to all cases, whether the spinal cord has suffered from concussion, or from the pressure of displaced vertebræ." 136.

*Affection of the Respiration.*

If the cord be lacerated or divided above the origin of the phrenic nerves, inspiration can no longer take place, and the animal dies instantly. Artificial respiration prolongs life for a few hours. Pressure on the superior portion of the spinal cord, in consequence of dislocation or fracture, is attended with a similar result. Destruction of the transverse ligament, allowing the odontoid process of the axis to press forward on the cord, has in several instances suddenly killed the patient. But this is not universally the case. A remarkable instance of this description is contained in the present volume of the Society's Transactions. It was presented to the Society by Mr. Phillips, and we shall introduce it here.

*Case.* In the Summer of 1827, William Cross, an agricultural labourer, was standing on a hay-rick, when he slipped off it, and fell to the ground head foremost, the occiput coming in contact with the soil. He was "stunned" by the fall, but was soon able to walk a distance of half a mile to the parish surgeon, by whom he was bled and purged. On the following day he felt scarcely any inconvenience from the accident, and in two days more he proceeded about his usual business. He now laboured under nothing but a "stiff neck."

Between three weeks and a month after the accident he was first seen by Mr. Benjamin Phillips. On that day the patient had walked two miles. Mr. Phillips examined the back of his neck. A small tumor was apparent immediately over the second cervical vertebra; pressure on it occasioned very little pain; nor was there any other lesion of sensation or of motion.

Mr. Phillips concluded that the disease was chronic scrofulous inflammation of the first or second vertebra, or both, and enjoined rest, leeches, and caustic issues. Between four and five months after Mr. Phillips first saw him, the following circumstances were observed.

"I noticed, what may have existed for some time before, a *thickness* of the voice, such as is produced by enlarged tonsils. I looked into the throat, and discovered that the tonsils were very large, but they did not afford any decided indication of recent disease. In a few days after I had discovered the change in the character of the voice, he complained of a little inconvenience in swallowing. I then carefully examined the throat, and discovered a slight projection or fulness at the back of the pharynx, as near as may be at the level of the body of the second cervical vertebra. It appeared to me that this circumstance was perfectly reconcilable with my diagnosis, and I concluded that an enlargement of the body of the axis had been produced, and that the interference with phonation and deglutition was so caused." 81.

Soon after this he had an attack of pleurisy, for which he was bled, and by which he was much debilitated. About nine months after the accident, anasarca came on, and in the forty-seventh week after the accident he died of hydrothorax. Up to the last week of his life, he was accustomed to walk to the water-closet, which was on the same floor with his bed, and was never assisted in taking food, even though lying on his back; and no evidence was ever afforded to those around him, that motion or sensation, with the exception already mentioned, was in the slightest degree impaired; nor had the difficulty of swallowing materially increased.

*Dissection.* All Mr. Phillips could obtain was permission to examine the back of the neck. He discovered that,—

"The condyles of the occiput still rested upon the articulating surfaces of the atlas, but the atlas was found to be, not a separate and independent vertebra, but an appendix to the axis. So much of its anterior portion, as includes the surfaces by which it is articulated with the occiput and with the axis, had been violently separated from the posterior portion of its ring, and had been carried in an oblique direction, downwards and forwards, until it arrived upon the same plane, but anterior to the axis, to the body and transverse processes of which it became attached by perfect bony union, whilst the posterior fragment had suffered no displacement.

Under these circumstances, the bone presented two spinal foramina, and four transverse, but no odontoid process. This organ having been fractured and separated no organ passed through the anterior spinal foramen." 83.

Mr. Phillips remarks:—

"In the present case, not only was there fracture and great displacement of the atlas, but a fracture and displacement of the processus dentatus, without any material disturbance of the functions of the economy. The blow was received upon the postero-superior surface of the occiput, the impetus was directed obliquely downwards and forwards in the course of a line, drawn from that point, as nearly as may be through the occipito-atloidal articulation. A portion of this ring of the atlas, immediately posterior to the articulating surfaces, is comparatively unsupported; this portion gave way, and the anterior portion was propelled downwards and forwards, between the axis and the pharynx, the posterior portion remaining as nearly as may be in situ.

The body of the atlas being thus displaced, one of two things was necessary, either that the odontoid process should be fractured, or that the transverse ligament by which it is attached to the atlas should be ruptured; in this case the former contingency obtained, and to this circumstance the patient owed his life." 84.

It does not appear that the medulla was compressed, although its direction must have been altered. The case certainly tends to shew, that severe injuries may be inflicted on this part of the spinal column, without lesion of the cord—and that the rule laid down by authors, " that a fracture of the processus proves instantly fatal, while a fracture of the cervical vertebræ (above the third), with considerable displacement, is almost immediately fatal," must be received with some degree of reservation.

We now return to Sir Benjamin Brodie. He relates a case, in which caries of the cervical vertebræ in a child proved fatal. The child died with symptoms resembling those of hydrocephalus. On examining the body, the ventricles of the brain were found to be much distended with fluid. The transverse ligament of the second cervical vertebra had given way, and the odontoid process formed a considerable projection into the spinal canal. The dura mater however was entire, and prevented the dislocation being so complete as it would have been otherwise. The pressure on the spinal cord was not sufficient to destroy its functions, although it might well be supposed to have operated as a cause of irritation, so as to produce the effusion into the ventricles of the brain. A boy, aged sixteen, died in the hospital under very similar circumstances.

When the cord is seriously injured below the origin of the phrenic nerve, and above that of the intercostal nerves, inspiration is performed only by the diaphragm, and expiration by the pressure of the viscera assisting the relaxation of that muscle, by the elasticity of the thoracic parietes, and by the elastic resilience of the lungs. Such respiration we may well imagine to be exceedingly imperfect. We observe that the patient is incapable of expectorating mucus if it be collected in the trachea: that, if he coughs, the cough is peculiar, being effected by a forcible inspiration, followed by a sudden relaxation of the diaphragm; and that if he be placed in the sitting posture, so that the pressure of the abdominal viscera is removed from the diaphragm, he breathes with much greater difficulty than when he is lying down. Recoveries in such cases are very rare. The patient seldom survives beyond the sixth day, and more frequently dies within forty-eight hours.

As these symptoms depend not simply on the situation of the fracture, but on the amount of lesion of the cord, they must obviously be regulated by that amount. In the first instance, the cord may not have been much hurt, and the symptoms may come on with its subsequent disorganization. In one case which occurred to our author, there was a fracture of the seventh cervical vertebra, followed by a softening and dissolution of the spinal cord; the difficulty of respiration did not take place until the twelfth day; but death occurred in less than three days afterwards.

Of course, the lower down in the back the injury inflicted on the cord, the less the interference with the respiratory function. Wherever, says our author, the injury is situated, a disposition to cough, with a copious expectoration, is likely to occur some time after the accident. In one case, in which there was a fracture of the eleventh dorsal vertebra, with softening of the spinal cord in the same situation, these symptoms began as early as the third day; and such was the disposition to cough, that it was induced by any slight change of position. Nevertheless this patient survived until the end of the fifth week.

It does not appear that in these cases of injury of the spine, the expired air has been submitted to chemical examination. It would not be uninteresting to ascertain if the changes occasioned by respiration in the air and blood, continue as before. It is not likely that there should be any material difference, as we must presume the consequences of the lesion of the cord to be simply the imperfection of the mechanical act of expansion and contraction of the thorax. After tying the pneumo gastric nerves, the arterialization of the blood is greatly impaired.

*Priapism.* "This is a very common symptom of injury of the spinal cord, and it is remarkable that, although under ordinary circumstances the erection of the penis is the result of an impression communicated from the sensorium, I have never known it to occur, in these cases, except in combination with paralysis. Priapism may take place whether the patient suffers from the effects of simple concussion of the spinal cord, or from those of pressure. It seems to be connected with injuries of the upper, rather than with those of the lower portion of the cord: at least, I am not aware that I have met with it where the seat of the injury has been below the sixth dorsal vertebra. It is for the most part an early symptom, shewing itself in the course of the second or third day, and it seldom continues after the first fortnight. It occurs even where the sensibility of the parts is totally destroyed, and may be induced by the mechanical irritation caused by the introduction of the catheter, where the patient is entirely unconscious of the operation. This circumstance was pointed out to me many years ago by Professor Macartney, of Trinity College, Dublin; and I have had many opportunities of verifying the correctness of the observation." 141.

The occurrence of priapism after the total abolition of sensation and of voluntary motion in the neighbouring parts, is a good instance of the continuance of the "reflex function," after the communication with the encephalon has been destroyed. In the state of health, priapism may result either from an impression of which the sensorium is conscious, or from one upon the nerves of the genito-urinary system, of which it is not conscious. Instances of the latter are not unfrequent. Irritation of the rectum, of the neck of the bladder, the introduction of an instrument, will occasion priapism almost without the consciousness, certainly against the will of the individual. The erections that occur in sleep, are frequently unattended with consciousness, and, resulting as they usually do, from fulness of the generative system, must depend essentially on the operation of the reflex function of the medulla.

*Affections of the Urinary Organs.*

These are extremely important, on two accounts—first, because they tend to abridge the patient's life; secondly, because they require the assistance of the surgeon.

Paralysis of the lower half of the body is attended with an inability to void the urine. Sometimes the patient is conscious in some degree of the retention—sometimes he is not at all so. If the catheter is not employed, the urine flows involuntarily, as in other cases of over-distended bladder. At other times, the urine dribbles away, although the bladder is contracted. Retention of urine is an early symptom—continues to the last in fatal cases—and, where recovery takes place, disappears earlier than the palsy of the limbs. The following account of the changes which occur in the urine, and in the urinary organs, is too concise to be condensed, and too valuable to be omitted. Long as the extract is, we give it with pleasure.

"The first effect of a severe injury of the spinal cord is not unfrequently to occasion a marked diminution in the quantity of urine secreted. This is most observable where the injury is in the lower part of the neck, and where, in consequence, the function of respiration is very much impaired. Thus, in a patient in St. George's Hospital, in whom there was a forcible separation of the fifth and sixth cervical vertebræ, complicated with fracture and depression of bone, and laceration of the spinal cord, four ounces of urine were drawn off by the catheter at the end of twenty-four hours after the occurrence of the accident, and when he died at the end of twenty-six hours more, the same quantity was found in the bladder, none having been voided in the intermediate time. The same thing, however, may occur when the injury is in the lower part of the spine. For example: a gentleman received a blow on the loins, which occasioned, in the first instance, a partial paralysis of the muscles of the lower extremities. He never experienced any difficulty in voiding the urine, but the secretion was almost entirely, if not entirely, suspended during the first twenty-four hours. I did not see this patient at the time, but I cannot doubt the accuracy of the report which he made when I was consulted some time afterwards.

In some cases, the urine which is first secreted after the occurrence of the accident, although of an acid quality, and free from mucus, has a peculiarly offensive and disgusting odour. In other cases the urine is highly acid, having an opaque yellow appearance, and it deposits a yellow amorphous sediment. In one case this colouring matter was in such abundance that it was found after death to have imparted a yellow tint to the mucous membrane of the bladder, which at the same time bore no marks of inflammation, even exhibiting less appearance of vascularity than under ordinary circumstances.

But the most common change produced in the urine by an injury of the spinal cord is the following. It is voided of an ammoniacal odour, and turbid; when allowed to cool and remain at rest, it deposits a large quantity of adhesive mucus, and when tested with reddened litmus or turmeric paper, it is found to be highly alkaline. After some time a quantity of white matter (phosphate of lime) may be detected in the mucus, and it is tinged with blood. At a still later period a considerable quantity of coagulum of blood is blended with the mucus and urine. These appearances very commonly shew themselves as early as the second or third day after the occurrence of the accident; sometimes not before the end of a week, or even eight or nine days. I have not observed that injury of one part of the spine is more liable to produce them than injury of another. There is a great variety as to the period of their duration. In fatal cases they sometimes continue to the last, even though the patient should survive for several weeks, or even months; at other times they continue for two or three weeks, then subside, and the urine remains transparent, and of an acid quality afterwards. In other cases the quality of the urine varies almost from day to day, without any manifest reason for the change. It may be alkaline, depositing adhesive mucus; then clear and acid; then alkaline again; and these alterations may take place several times in the progress of the same case.

It is well known to pathologists that such adhesive mucus as is here described is never a constituent part of the urine as it is secreted by the kidneys, but that it is furnished by the mucous membrane of the bladder and ureters, especially of the former, when in a state of inflammation. The effect therefore of an injury of the spine is, in many instances, to occasion inflammation of the mucous membrane lining the urinary organs; and the consequences of such inflammation, where the urine has continued alkaline and loaded with mucus up to the time of the patient's death, are very manifest on dissection. The mucous membranes of the bladder, ureters, pelvis, and infundibula of the kidneys, is highly vascular; and in some cases the bladder is lined with phosphate of lime, which the mucus has deposited on its surface. Occasionally spots of extravasated blood are found in the glandular substance of the kidneys, and loose masses of coagulum in the pelvis of the kidneys and in the bladder.

An interesting question here presents itself, whether the inflammation of the mucous membrane of the bladder be a primary or a secondary disease? Whether the injury of the spinal cord operates directly on the mucous membrane, or whether its first effect is to alter the quality of the urine, as it is formed by the kidneys, the mucous membrane becoming affected afterwards, in consequence of the irritation excited in it by the contact of an unhealthy and stimulating secretion? These points remain to be determined by future observations." 145.

*Affections of the Digestive Organs.*

In the first instance, whatever be the seat of the injury in the spinal cord, the bowels are torpid, so that they cannot be made to act except under the influence of the most powerful purgatives. Then the abdomen becomes tympanitic; and in fatal cases, however protracted, these symptoms generally continue in a greater or less degree to the last. The fæces are passed without the patient's consciousness. In many cases in which the injury is in the cervical portion of the spinal cord, and death takes place in the course of two or three days, there is a disposition to vomit. In one case which fell under Sir Benjamin's observation there was incessant vomiting of a large quantity of dark-coloured fluid: in another case, in which the patient died on the fifth day, during the two days which preceded his dissolution there was a perpetual gulping, and ejection of a similar diseased secretion.

In more protracted cases the alvine evacuations are of a black colour; semi-fluid, somewhat resembling tar or treacle in their appearance, and of a peculiar and offensive odour.

"It has not been ascertained," continues Sir Benjamin, "as far as I know, what is the nature of the substance on which this dark colour depends. It certainly is not mere extravasated blood. Probably it is furnished by the secreting vessels of the stomach and intestines, and corresponds to the dark-coloured matter which is sometimes vomited at the termination of typhus fever, or to the black sordes which, under the same circumstances, are attached to the gums and teeth. It is only within the last two or three years that my attention has been directed to the subject, and further observations are required for its elucidation." 146.

*Gangrene.*

The disposition to gangrene in the parts below the seat of injury is well known. It is evidently the result of the diminished nervous influence. In many cases, in which the injury has affected the cervical portion of the cord, sloughs begin to be formed, not only on the sacrum and nates, but even on the ankles, as early as the second day. When the injury is in the dorsal part of the cord, the sloughs are generally, but not always, confined to those parts on which the pressure is greatest.

*Affection of the Sensorium.*

"I have seldom," says Sir Benjamin, "observed the sensorium to be materially affected, except where the injury was in the cervical portion of the spinal cord, and here the results are very different in different cases.

Thus, in one patient, in whom there was a fracture of the fifth and sixth cervical vertebræ, with displacement of bone and laceration of the spinal cord, the functions of the sensorium were in no degree disturbed, the patient being perfectly conscious and talking rationally. Another patient, in whom the same part of the cord was bruised and lacerated, became comatose soon after the accident. On some blood being taken from the arm, the coma subsided; but at the end of twelve hours he became again comatose, and continued so until he died, ten hours afterwards. A third patient, in whom there was a fracture of the fourth and fifth cervical vertebræ, with softening of the spinal cord, was at first perfectly conscious and sensible. In less than twenty-four hours he fell into a state approaching to that of complete stupor; then became delirious, and continued so until he died, thirty-six hours after the accident. A fourth patient, in whom there was a small extravasation of blood in the centre of the spinal cord, opposite the fifth and sixth cervical vertebræ, died in less than forty-eight hours, having been sensible and conscious nearly to the last, but the pupils of his eyes being contracted."

*Symptoms not included under the foregoing Heads.*

A. The first effect of any severe injury of any portion of the cord, is a greater or less degree of collapse. Occasionally there is a rigor soon after the accident.

B. When the injury is in the lower part of the neck, the patient, our author proceeds to state, not unfrequently dies before complete re-action is established, the pulse remaining feeble to the last; or it may beat distinctly, but not oftener than fifty or sixty times in a minute. In the majority of cases, however, after the first twenty-four hours, the pulse rises to ninety-six or a hundred in a minute; but still it is feeble and contracted, indicating a state of great general debility rather than the existence of an active inflammatory disease. The appearance of the tongue corresponds to the character of the pulse; and it is not unusual at the end of twenty-four hours to find it dry and parched, covered with a brown fur, which is soon converted into a black crust, resembling what we observe in the last stage of a continued fever.

C. When the injury is in the lower part of the neck, but not such as to occasion death in the first three or four days, or where it is in the dorsal or lumbar regions, and the patient does not die early, or ultimately recovers, the pulse usually remains for a considerable time more frequent than natural, varying from ninety to one hundred and twenty beats in a minute, but feeble and contracted, the tongue at the same time becoming more clean and moist than it was in the first instance. If blood be drawn under these circumstances, the coagulum is of a large size and loose texture; often never exhibiting any inflammatory appearances whatever; sometimes having a slight buffy coat on its surface in the first instance, but none afterwards. These observations apply to the cases in which softening of the cord takes place, and offer, Sir Benjamin Brodie thinks, an additional reason for supposing its softening after injury not to be inflammatory.

D. Inflammation of the membranes of the cord is less frequent after injuries of the spine than inflammation of the membranes of the brain is, after injuries of the head. In the cases which Sir B. Brodie has seen, the inflammation of the membranes and the softening of the cord have gone on simultaneously. Superadded to the ordinary symptoms of the latter, there were profuse perspirations, and severe and repeated rigors, marking the occurrence of suppuration. There were also spasmodic twitches of the voluntary muscles, which our author attributes to the pressure of pus upon the cord.

In reference to this subject, we may introduce a passage from Dr. Hall. He speaks thus of the symptoms of inflammation of the membranes of the spine.

"Amongst the first symptoms of spinal *meningitis* is local *pain* in some part of the spinal column, augmented by the movements of the patient, and by percussion, but rarely, if ever, by pressure, along the spine. This pain sometimes extends along the back and limbs, in which there is then tenderness on pressure, —a symptom which may serve to distinguish meningitis from myelitis, in which there is usually loss of sensibility.

The next important symptom is spasm, or various kinds of muscular contraction. The head, the neck, or the trunk, is bent backwards; or there is trismus, torticollis, partial or complete opisthotonos, or contractions of the limbs,—constant, or recurrent, or exacerbated, in paroxysms, on moving, or being moved, &c. with extreme pain. Sometimes there are convulsions." 136.

The phenomena of tetanus and epilepsy, of the convulsions of children, of hysteria, and of chorea, would seem to shew how little organic lesion is absolutely requisite for the production of spasmodic contractions of the muscles. This remark of Sir Benjamin Brodie's should lead to a more precise investigation, than, probably, has hitherto been prosecuted, of the actual causes of spasm after spinal injuries.

E. "Sir Charles Bell has described two cases, in which inflammation and suppuration of the membrane of the spinal cord followed injuries of the spine, unconnected with softening of the cord itself, and producing symptoms a good deal similar to those which are described as taking place where these membranes are inflamed from other accidental causes. In the first of these was a fracture of the eleventh dorsal vertebra. The patient was affected with delirium, attended with a rapid pulse. The most remarkable circumstance was, that there was no loss either of sensation or of the power of voluntary motion. Death, preceded by typhoid symptoms, took place on the fifth day.

In the other case recorded by Sir Charles Bell, the injury was in the lower cervical and first dorsal vertebræ, and the whole of the spinal cord, from this part to the lower part of the loins, was found after death to be bathed in pus. There were no severe or urgent symptoms for the first eight days; then the patient was seized with violent convulsions, followed by fever and delirium. There were no paralytic symptoms until the tenth day, when there was a difficulty in raising one arm, and this was followed by complete paralysis of the lower limbs two days afterwards. Death, preceded by typhoid symptoms, took place nearly three weeks after the occurrence of the accident." 153.

Sir B. Brodie has seen no cases precisely similar to these. He relates one of some interest. A patient had fracture of the sixth dorsal vertebra, and the usual symptoms. On the tenth day gangrene commenced upon one foot, and over the os sacrum. The sloughing rapidly extended, and the greater part of one glutæus maximus came away. On dissection there was softening of the cord in the middle of the back. The sloughing had extended to that part of the spinal canal, contained in the sacrum, and lumbar vertebræ, between which and the dura mater much pus had been deposited. Between the dura mater and the arachnoid was a layer of yellow lymph, most abundant in the situation of the cauda equina, but extending as high as the lower dorsal vertebræ. From the latter to the middle of the back, both the cord and its membranes were free from disease.

F. A review of the consequences of injuries of the spinal cord establishes the following general conclusions; *that,* laceration, compression, or softening of the cord leads to no material difference either in symptoms or results; *that,* the great majority of the symptoms are the same, whatever the part of the cord injured; *that,* the lesion of the respiratory function must be excepted, in consequence of its being regulated by nerves coming off from a particular region, and *that,* of course, the parts immediately affected with paralysis must, for the same reason, be excepted too; *that,* a consideration of these two circumstances explains the increase of danger, in proportion as the lesion is higher in the cord.

Cases of recovery, observes Sir Benjamin, in concluding this history of the symptoms, even after what may be regarded as a severe injury of the spinal cord, are by no means uncommon. If it suffers from the effects of concussion, the recovery may be complete; the period of recovery varying from three weeks to twelve months or more. If the cord be lacerated, or much compressed by displaced bone, the patient may live, but without recovering from the paralysis. Under these circumstances, life may be prolonged for an indefinite period. A man was admitted into St. George's Hospital, in January, 1823, who had met with an injury in the preceding August, in consequence of a mass of chalk having fallen upon him while working in a chalk-pit. Mr. Hardwicke, of Epsom, being sent for, found the first lumbar projecting over the last dorsal. With some difficulty he reduced the displaced vertebra to its natural position, the reduction taking place with a jerk or snap. At the time of his being received into the hospital, he had some power of using his lower limbs while in bed, but he could neither walk nor stand, and he was unable to empty his bladder without the aid of the catheter. He remained nearly in the same state when he quitted the hospital, two months afterwards. Two or three years after that he was still alive, and without any material alteration in his symptoms.

### TREATMENT OF INJURIES OF THE SPINE.

1. If a vertebra or vertebræ should be dislocated, or displaced and fractured, the first question which presents itself, is the propriety of attempting to restore it to its natural situation. Both reasoning and facts concur to recommend an attempt of the sort, if conducted with caution. When not made cautiously, we may easily conceive what consequences may ensue, and Boyer states a case in which a child died under it.

In displacement of the cervical vertebræ, the greatest care is requisite; reduction is less hazardous, and will, probably, be more successful when the lumbar are concerned.

"In proof of this assertion, I may refer to a case which I have already described, which occurred in the practice of Mr. Hardwicke, of Epsom; the patient being afterwards admitted into St. George's hospital, labouring under paralysis of the lower limbs. In another case, to which also some allusion has been already made, and which occurred under my care in St. George's hospital, there was a fracture with great displacement of the third and fourth lumbar vertebræ. When the patient had recovered from the state of collapse which had followed the first shock of the accident, I endeavoured, by fixing the thorax and cautiously extending the pelvis, to restore the vertebræ to their proper place. The attempt was in some degree successful, and no ill effects of any kind resulted from it." 159.

2. From the analogical instance of the fracture of the cranium, it has been proposed, in cases of fracture and depression of the arch of the vertebræ, to apply the trephine and elevate the bone. The following are the reasons of Sir Benjamin Brodie, for disapproving of the operation.

"The question respecting such an operation seems to me to lie in a very small compass. If the whole or nearly the whole of a vertebra be driven forwards, the depression of the posterior part of it will of course occasion a diminution of the size of the spinal canal; but the removal of any portion of the vertebra which is accessible to an operation, will be of little avail, as the irregularity in the anterior part of the canal, made by the displacement of the body of the vertebra, must be the same after, as it was before, the operation.

If there be simply a fracture on each side of the spinous process, with a depression of the loose or intermediate portion of bone, of course there must be a corresponding diminution of the size of the vertebral canal; but as that canal is much larger than the spinal cord, which it contains, it does not follow that the spinal cord is really compressed, or that any material diminution of the symptoms would follow the elevation of the depression.

But let it be supposed that the spinal cord is really suffering from pressure: it has been already shown, that a much less degree of violence than that which is necessary to occasion a fracture of the spine, may produce concussion, softening, and ultimately dissolution of the spinal cord, with a train of symptoms much worse than those which arise from simple pressure. Now no operation can be of the smallest advantage in this respect: but, on the contrary, if it be necessary to apply the saw in the performance of it, the jar and disturbance of the parts which this must occasion is even likely to aggravate the mischief.

If these views be correct, it is evident that the cases, in which there are any reasonable grounds for the performance of the operation, must be of very rare occurrence, and that even under the most auspicious circumstances it must be doubtful whether it may not be productive of harm rather than of good to the patient.

Nor, as far as I am acquainted with the results, do the experiments, which have been hitherto made on the subject, lead to any more satisfactory conclusion. I am not aware that in any of the cases, in which it has been hitherto performed, the operation has proved the means of preserving the patient's life, or even of relieving any of the more important symptoms." 161.

The proposal of trephining the spine, has certainly not advanced in favour with the profession, since the performance of one or two operations in the Borough Hospitals. The arguments against it may be resolved into these simple propositions :—*that* we operate in ignorance of the amount of injury inflicted on the vertebræ—*that* we are also ignorant of the amount of mischief inflicted on the cord—*that*, consequently, the operation may not be applicable to the state of the bones, nor beneficial to that of the cord—*that* the operation itself is a severe one, and if it does no good, will probably do harm. The consideration of all these circumstances, throws a great degree of doubt on the propriety of the operation, and not a small one on its safety.

The very nature of the arguments against the operation, would recommend it under certain circumstances, *supposing* ourselves equal to their determination. If a surgeon could be satisfied from the mode in which the injury occurred, or from any features in the case, that the injury of the bone was limited to the arch, and that the cord was sound, or, if the amount of probabilities was such as to render that conclusion highly reasonable, we suppose he would be justified in using the trephine. But to leave a question in such a state, is virtually to decide it in the negative, and we may be sure, in this instance, that the cases adapted for an operation will be very few, while the responsibility incurred by the surgeon will render those in which it is performed still fewer.

3. Absolute repose in the horizontal posture, is an item of treatment too obvious to detain us.

4. "I have show that, in some cases, an injury of the spine is followed by inflammation of the membranes of the spinal cord, and there can be no doubt that it is then necessary to take blood from the arm, and even to repeat the blood-letting several times. The state of the pulse forms a sufficient indication to the practical surgeon of the necessity of such active treatment, and the appearances of the blood after it is drawn will assist his judgment in determining to what extent it should be carried. It is, however, if my experience has not much misled me, a great mistake to suppose that blood-letting is always proper. In the majority of cases the state of the pulse is such as actually to contra-indicate the abstraction of blood, and as I have already stated, the blood when drawn does not in general present those appearances which are supposed to mark the existence of inflammation. I have no reason to believe that blood-letting arrests the process of softening and dissolution of the spinal cord, and indeed I have usually found that the symptoms which mark the existence of these changes make a more rapid progress, in proportion as a larger quantity of blood is taken away. The weak and contracted pulse, the disposition to gangrene, the alkaline quality of the urine, the black and offensive alvine evacuations, and the brown or black fur on the surface of the tongue, would, under other circumstances, be regarded as proofs of depression and debility, indicating the use of stimulants rather than of what are called antiphlogistic remedies. From all that I have seen, I cannot doubt that much harm has arisen from the indiscriminate application of the practice, which is usually proper and necessary after injuries of the head, to cases of injury of the spine." 162.

5. The torpor of the bowels, and the bad state of their secretions, conspire to demand purgatives. The addition of some stimulant, as ammonia, assafœtida, &c. to them is of service.

6. The catheter is required from the commencement. When the urine becomes alkaline, and the bladder secretes adhesive mucus, the latter should be emptied several times daily, and in some cases it is useful to wash it out with injections of warm water.

7. The treatment of the gangrene that succeeds spinal injuries is any thing but satisfactory. The prevention, as far as possible, of pressure, and the exhibition, in a judicious manner, of stimulants, is all that we can do, which is very little.

Here we part from the subject of injuries of the spine, and from Sir Benjamin Brodie. This paper, like all his others, is characterized by an exact and inductive spirit, the absence of hypothesis, and the caution so indicative both of knowledge and philosophy. It is by such papers, and by the kind of observation they are founded on, that surgery will be essentially advanced, and rendered as little an empirical art, and as much a demonstrative science, as the nature of its objects will permit.

We must quit the pathology of the spinal cord. The lengthened analysis of Sir Benjamin Brodie's valuable paper precludes our adverting to the idiopathic diseases of the medulla and its membranes, to the consideration of which we shall return hereafter.

The works of Dr. Hodgkin and of Dr. Carswell claim what amount of space remains, and to these we shall proceed.

We gave a sketch of the contents of Dr. Hodgkin's work in the last number of this Journal. Of the twelve lectures which that work contains we noticed two. The ten remaining, are devoted to the arachnoid membrane—to the pericardium—to the pleura—to the peritoneum, tunica vaginalis, and bursæ—to parasitical animals—to adventitious serous membranes—to the general characters of malignant disease—to scirrhous and fungoid tumors—to the colours of animal tissues—and, lastly, to malignant disease in general.

### II. Parasitical Animals.

We shall, probably, select the subject of malignant diseases for an article in the succeeding number of this Journal. At the present moment we will pick out the lecture on parasitical animals, because some recent observations and researches have conspired to bring them rather prominently forward.

The lecture in question is the seventh. It occupies forty pages of the work, and some powerful compression must necessarily be exerted to reduce it to the limits we are able to afford it.

Dr. Hodgkin attempts little more than a classification and a description of the parasitical animals which infest man. Of these, some classes are found imbedded in the substance of, or lodged in, internal cavities; and are never, or, at most, but rarely and accidentally, met with out of the body. They constitute the intestinal animals of Cuvier, and the entozoa of Rudolphi. Other parasitical animals support a more distinct life, and have recourse to the animal which they infest only for their supply of food, and for the deposition of their eggs or larvæ. It is for a part only of their lives that they are imbedded in, or attached to, the animal, at whose expense they subsist. The greater number of these belong to the active class of insects; but a few, as, for example, the leech, are found amongst the annelides mollusca.

The entozoa are first considered. The question of their spontaneous generation we leave untouched. Probabilities are rather against than for it. The entozoa are found in the tissues as well as in the cavities of the body. They have no distinct organs of circulation, of respiration, or of innervation, and in other respects they present great varieties in their organization. They have been arranged under three heads: 1. The vesicular worms. 2. Flat worms. 3. Cylindrical worms. The two first, or the vesicular, and the flat, belong to Cuvier's order of parenchymatous intestinal animals, or those whose imperfect viscera penetrate and ramify through their substance, and those in which not even a trace of alimentary canal can be perceived. The cylindrical worms, on the other hand, are included in Cuvier's order of cavitaires; or those which have an intestinal canal, furnished with a mouth and anus, and floating in a distinct abdominal cavity.

1. *Of Vesicular Worms or Hydatids.*

"The vesicular worms are possessed of a caudal bladder or vesicle, which constitutes the greater part of the animal, and on which the name of the order depends. It is of a greater or smaller size; and is either proper to one individual, or common to several. In one genus, the acephalocystis, the animal consists of nothing but this bladder. Others possess a body, which is always very small in proportion to the animal, and a head bearing some resemblance to that of the tænia, and variously armed with tentacula and sucking orifices." 183.

The vesicular worms are not very rare in man—they infest all portions of his body—interfere with his existence—and have been found at all ages, even in the fœtus. They are met with in many of the mammalia, and even in cold-blooded animals. Dr. Hodgkin agrees with those who regard all these cysts as possessed of some degree of vitality. He adopts the classification of Cloquet, Portal, and Beclard, which enumerates their genera, as 1. the Cysticercus; 2. the Polycephalus; 3. the Diceras; 4. the Echinococcus; and 5. the Acephalocystis.

A. *Cysticercus.* It has a head somewhat resembling that of the tænia; and a nearly cylindrical body, terminated by a caudal vesicle. It generally exists singly in the cyst which contains it. It is more frequently met with in the inferior animals, than in man; and is by no means rare in the livers of sheep.

It appears to exist in a specimen of human lung at Guy's Hospital. Dr. H. has seen it taken from the pia mater—and he has seen it in the cellular membrane of the thigh. Dr. Sharpey says that cysticerci are not very uncommon in some parts of Germany.

The cysticerci of different animals differ in their form and proportion: those in man have, generally, the neck about equal to the diameter of the vesicle, from which it proceeds with a gradual diminution in size. They are frequently found in the liver, and even in the brain of sheep. The trephine has been successfully employed in these animals.

"The enclosing cyst in which the cysticerci are lodged, whether derived from cellular membrane alone or from a serous membrane in conjunction with the cellular, is generally thin; but when, in consequence of the death and contraction of the cysticercus, the enclosing cyst is allowed also to contract, it may acquire considerable thickness, and a dense structure. This is very conspicuous in the livers of sheep, in which the whole sac sometimes acquires a bony character. The death and contraction of the cysticercus constitute the process by which a natural cure is effected." 186.

Passing over the genera Polycephalus, Diceras, and Echinococcus, we stop at the Acephalocyst.

B. *Acephalocystis.* This is far the most frequently found in man. Hydatids of this kind are enclosed in a cyst which is apparently derived from the cellular tissue of the surrounding parts; but acquires a dense and almost semicartilaginous structure, in which bony matter is sometimes deposited. This cyst, in some very rare cases, is said to have been absent, an assertion of which our author seems doubtful. We shall present his account of the structure and character of the acephalocysts, as it is concise and pretty complete.

The proper hydatid cysts, which have a lamellar structure, and in consistence bear a great resemblance to coagulated white of egg, have not the slightest connexion with the enclosing cyst; and between them, there is generally a thin and unequal layer of a dirty, yellow, semi-concrete substance, which may possibly be an excrementitious secretion from the hydatid itself. The proper cyst is filled with a fluid; which, though generally clear and limpid, is sometimes turbid and discoloured. On the whole, this fluid contains a very minute quantity of animal matter.

Though a large acephalocyst is sometimes solitary, more frequently it contains a number of smaller cysts, which may themselves hold others smaller still.

"The young hydatids appear to be produced from different parts of the parent cyst; to which they are described, by authors, as being found attached, sometimes on the inside, and sometimes on the outside. For my own part, I have never known an acephalocyst to produce smaller ones, except from its internal surface; and to this I have not seen them attached, except when of almost microscopic size. Whilst scarcely a few hundredths of an inch in diameter, they may be seen in great numbers, perfectly detached, in the fluid of the parent; and neither then, nor when of larger size, have I been able to discover the least trace of neck, or other attachment, to the superior cyst. On the inner surface of the latter there are frequently seen numerous elevations, variously disposed, and differing both in form and magnitude, but generally minute. The opaque white spots affecting the hydatid membrane are not confined to the internal surface: they are occasionally found upon the external surface. I have seen them half or two-thirds of an inch in diameter, of a circular figure, with an irregular surface, something like that of a cauliflower. It is well to be acquainted with the appearance of these spots; since it may happen that an hydatid tumor may be spontaneously evacuated by a communication with some part of the alimentary canal, and the dead membranes may become so much altered as to be scarcely recognizable whilst coming away in fragments. Yet should one of these fragments possess one or more of these elevations, it is so characteristic, as to remove all doubt respecting the nature of the case. These inequalities have been supposed to depend on nascent hydatids; and the differences which they present have been assumed to be the characteristics of three species of acephalocystis. I am satisfied that many of these elevations are morbid growths from the parent cyst; and others of them possibly point out the spots whence the young have already been detached. I am not however prepared to affirm that none of these elevations are attributable to nascent hydatids. In a case examined by Francis Sibson, jun. and myself, numerous transparent spherical granules were seen in the parietes of a parent hydatid acephalocyst: they were, in all probability, nascent young ones, and appeared to throw some light on the mode of production. They were situated much nearer to the internal than the external surface; and, although a few of them were of a larger size than some of the small, perfectly-formed, detached hydatids which in other cases I have seen within the interior of the parent, in this instance there were none which had become detached. I imagine that the separation or retention of these little bodies may in some degree depend upon the nature of the internal surface of the parent. If this internal surface be formed by an extremely thin membrane, or by a scarcely concrete substance, they may become detached almost as soon as formed; but if the internal surface be composed of a firm membrane, or some tenacious substance, they may be retained, as in the instance last referred to, and even acquire the semblance of a peduncular connexion amongst themselves or with the parent cyst." 189.

The junior cysts are similar in structure to the parent, and are usually spherical. But Dr. Hodgkin has seen them present an hour-glass contraction, and, on one occasion, opaque white lines, which, after pursuing a straight course, bifurcated once or twice at similar angles.

"Some of the secondary acephalocysts are slightly milky or opaline: others are perfectly transparent, and capable of answering completely the purpose of a lens. They occasionally appear to have been produced at different times, or to have advanced with different degrees of rapidity. By the united effect of the growth of these cysts, the parent cyst is finally burst, and dies.

Amongst a collection of hydatids, it is by no means uncommon to find some which appear to have lost their vitality, and to have become collapsed, more or less opaque, and of a yellowish colour. These parasitical animals are supposed to enjoy but a short period of life. The hydatids of pigs and sheep are said to be produced in spring, and to die the following winter. It is certain, however, that in man, either the continued life of individual hydatids, or a succession of generations, will protract, not merely the existence, but the growth of hydatid tumors to a much longer period." 190.

Dr. Hodgkin relates a case corroborative of many of the preceding observations. He does not think that the mere existence of a solitary cyst affords a sufficient basis for separating it from the social one.

An interesting, but not a very promising part of the inquiry into the history of acephalocysts, relates to the possibility of removing them. Nature does so in two ways: 1st, By forming a communication between the cyst containing the hydatids, and either the surface of the body, or the intestinal canal, or some other cavity, as, for example, the urinary passages, by which they may be evacuated. Or, 2dly, the animal dies: its fluid is absorbed; and its membrane is folded and shut up, as an inert foreign body, within the outer cyst which contracts upon it. The ossification of the cyst, thought by some to be a natural mode of effecting the death of the hydatid, is looked on by Dr. Hodgkin as a result of it, a more probable opinion. In the liver, he believes the bile to be employed by nature for effecting the death of the acephalocyst. In this, we conceive, the doctor is rather confounding an accidental circumstance with a natural intention. If a parasitical animal is developed in an organ, the secretion of which is poisonous to it, and, if in the usual changes of the tissues which a foreign body occasions, the secretion does arrive at it, that must be looked on as a mere contingency, not a special effort of the natural powers. There is very little more intention in this mode of killing the parasite, than if it tumbled into a jar of aquafortis.

In some cases hydatid tumors have been cut into, and their contents thus got rid of. The extraction of the containing cyst is an important element in the cure.

"I have heard that some remarkable cases of abdominal tumor dependent on hydatids have been successfully treated by Dr. Recamier, one of the physicians at the Hôtel Dieu. From symptoms, with which I am not acquainted, he was induced very confidently to diagnose the existence of hydatids; and he steadily persevered in directing the repeated application of caustic, until the sac was opened, and the acephalocysts allowed to escape. Hydatids have been evacuated, and the patient cured, under the influence of a saline purgative: and Laennec, having noticed that sheep affected with vertigo dependent upon hydatids of the genus cysticercus are cured by feeding in salt marshes, has been led to recommend baths and fomentations containing a solution of muriate of soda, in cases in which the presence of hydatids has been known or suspected. He supports his views by the detail of several successful cases; yet the efficacy of this mode of treatment must so much depend on cutaneous absorption and transudation through the subjacent tissues, that, notwithstanding my veneration for the author, I cannot but be sceptical with regard to it. The diffusible nature and the anthelmintic virtues of turpentine, and of Deppel's oil, might entitle them to a trial, both internally and externally. It might also be made the subject of experiment on inferior animals affected with hydatids, how far the simple acu-puncture, or the more powerful electro-puncture, might succeed in procuring the death of the hydatids, and the cure of the affected animals without the expulsion of the cysts." 196.

An operation for the evacuation of the contents of a hydatid tumor should only be attempted under favourable circumstances. Dr. Hodgkin has seen two cases, where, after the death of the hydatids, and condensation of the inclosing cyst, inflammation and suppuration have occurred in the surrounding tissues, and fatal consequences have ensued. In both cases the liver was the seat of the disease.

### 2. *Of Flat Worms.*

The most remarkable of these are the bothriocephalus and the tæniæ. We need not enter upon their description. The following circumstances connected with the tape-worm are not unworthy of attention.

"I believe," says our author, "that few persons are aware of the remarkable appearance which the tænia assumes in its most contracted state. I had no idea of it myself, until I received an interesting communication on the subject from Dr. Barlow of Bath. He informed me, in a letter dated the 25th of 10th month (October) 1830, that, about a year before, after his usual visit to the United Bath Hospital, he was about to leave the establishment, when one of the nurses hastened to shew him a worm which had just been passed in a stool by a patient affected with acute rheumatism: it was about five inches long, of a dark colour, and marked by numerous rings. Not recognising any thing which he had ever before seen, he directed it to be set aside, until he had an opportunity to examine it. It was, in his presence, put into one of the porringers used for bleeding, together with a little water to prevent its becoming dry; and then committed to the care of the apothecary's assistant. The next day, on calling for the porringer, he was not a little astonished to find, instead of his acquaintance of the preceding day, a large mass of unfolded tape-worm, of considerable length, white, flat, jointed, and in every respect such as patients affected with tænia so often present to their medical attendants after the successful exhibition of a vermifuge. Though the Doctor was not aware that he had ever met with a description of the tænia in the state in which the one just mentioned was shewn to him, he supposed that it might have been described by elminthologists; but had sought in vain for such a description, when he sent his account to me. He was therefore induced to believe that the living tape-worm possesses the form and characters of the worm just described, as it was first seen; that its evolution is consequent on the death of the animal; and that naturalists generally have been led into error, by taking their accounts from the worm when dead, in which state it is almost always seen." 199.

Dr. Hodgkin, however, is for several reasons inclined to conclude that both the contracted and elongated states are natural to the tape-worm, and not post-mortem phenomena. The tæniæ appear to prevail in certain districts. They infested our troops stationed in and near the Cape.

3. *Of Cylindrical Worms.*

These possess a higher organization than the preceding. They have an abdominal cavity, an intestinal canal, a mouth and anus, and distinct sexual organs.

The genera to which our author adverts are the ascaris, the oxyuris, the trichocephalus, the filaria, and the strongylus.

Of the *ascarides* one species only is met with in man. The ascaris lumbricoides, or the lumbricus, as it is vulgarly called, is found, without any manifest difference, in man, the horse, the ass, the zebra, the quagga, the ox, and the pig. Generally speaking, it would appear that the size of these worms bears some relation to that of the individual in whom they are found. They sometimes exist in very great numbers. Dr. Hodgkin saw between fifty and sixty in the small intestines of a boy who died in La Charité of fever.

The ascarides lumbricoides are usually met with in the small intestines. At times, however, they are either carried or make their way into the large intestines, and are voided with the stools. At other times, in opposition to the peristaltic action of the intestines, they gain the stomach; and have even been known to make their exit from the mouth or nostrils. Instances are more rare in which these worms have escaped from the body by preternatural openings.

Sometimes the presence of these worms is not characterized by any definite symptoms. At others, there is the usual evidence of irritation of the intestinal canal. They are attended with an abundant secretion of unhealthy mucus, which is often thick, viscid, and opaque, and something like thick milk. "Broussais," says our author, "lays particular stress on the necessity which there is for a morbid, and, as he believes, an inflammatory state of the intestinal mucous membrane, as an essential condition in the propagation of intestinal worms. I have heard him, in his Lectures, interdict anthelmintics; and order that the gastro-enteritis should be treated, as the worms would be unable to exist in the healthy mucus!"

The *oxyuris vermicularis,* formerly termed ascaris vermicularis, particularly infests children, not unfrequently adults, is present in great numbers, and has its usual residence in the large intestine, especially the rectum. It occasions itching and irritation at the anus, sometimes about the neck of the bladder, and has been said to give rise to satyriasis. Leaving the intestinal canal, it may visit the pudendum, or even quit one person for another. Oxyures infest rabbits, mice, and horses.

The *trichocephalus*, or, as it was erroneously termed, *trichuris*, is a round worm, about two inches in length, a variety of which is found in man. On the Continent, it would appear to be the worm most commonly met with in the human intestines. The large intestines, more particularly the appendix vermiformis of the cæcum, are their usual habitation.

The *filariæ* are another family of round worm, by more than one species of which man is infested. They have a long, slender, thread-like body, pierced before by a round mouth. They are principally met with in cavities which do not communicate externally—in the cellular membrane, in the substance of the muscles, and in the parenchymatous structure of the viscera. The most remarkable is the filaria medinensis, or guinea-worm, of which, from its celebrity, we need say nothing.

"Another species of filaria has been described as occurring solitarily in the lungs of persons labouring under phthisis, and is called the filaria bronchialis; or, by Treutler, who has given the principal description of it, the hamularia lymphatica; or, by Rudolphi, the hamularia subcompressa. It is an extremely rare worm; and, by some, the existence of such a species is doubted. However rare or doubtful the existence of filariæ may be in the lungs of man, worms, probably belonging to this family, are occasionally found, in great numbers, in the lungs of some of the inferior animals. I have seen very many of them in the lungs of two specimens of boa or python which died in this country. The lungs were at the same time very much affected with tubercles." 209.

Occasionally, though rarely, some other kinds of round worms are met with in man. Of these, the strongylus gigas is the most remarkable. It is described as the largest of all the intestinal worms; and is said to be two or three feet, or even more, in length, and as thick as one's little finger. The most frequent seat of its development is one or other of the kidneys, though it has been found in other organs. It is met with in different animals, as the dog, the wolf, the marten, and, at times, in man. When found, it is coiled up, distending the organ in which it is situated, and occasioning the destruction of its glandular structure. Strongyli of this species, but of small size, have sometimes passed off by the urethra. Mr. Lawrence has related in the Medico-Chirurgical Transactions the case of a female who voided, from time to time, many hundreds of small worms from the urinary bladder. It is not quite clear whether these were strongyli, or a new species which have been designated spiroptera hominis. The horse is subject to a species of strongylus, called equinus, which, penetrating to the arteries, causes aneurysm. We believe that the abdominal aorta is peculiarly subject to this kind of aneurysm.

"A small species of parasitical animals has lately been discovered affecting the muscles of voluntary motion, amongst the fibres of which they have been found generally dispersed over the body. They consist of very minute cysts, of an oblong figure, in size and colour bearing considerable resemblance to the young of pediculi attached to the hair of persons of dirty habits. When examined with a lens, these little cysts are seen, in many instances, not to be perfectly ovoid, but to be irregularly contracted towards one extremity, so as to form a sort of short and imperfect neck. It is also not uncommon to find some opacity towards one or both of the extremities. They are placed in the direction of the fibres; and are lodged in the cellular membrane immediately investing the muscular fibrillæ, or the tendinous fibres to which they are attached. When the specimen is sufficiently recent, one, and sometimes two thread-like worms may be discovered coiled up in each of the cysts. This little worm was first seen by that indefatigable and talented naturalist Richard Owen, who has minutely and carefully examined it, and who regards it as somewhat allied to the eels found in paste and vinegar. He has given it the name of trichina spiralis." 211.

The cysts were discovered and described before the worms that they contain were distinguished. The trichina appears to be confined to the muscles of voluntary motion, and to the tendinous fibres connected with them. In a case in which Dr. Hodgkin found them in all the voluntary muscles, even in the lumbricales of the foot, they stopped at the lower constrictor of the pharynx, and were not discoverable in the œsophagus, nor in any of the involuntary muscles.

No symptoms indicative of the presence of trichinæ have been hitherto made out. The cysts may become ossified, or earthy, probably when the worm has died and become invisible, for in such cysts Mr. Owen has not found the worm. In other cases, however, from some other causes, it has not been discovered.

"I must not quit the subject of entozoa without noticing the microscopic and very remarkable animalcules discovered in semen. They have been particularly investigated by Prevost and Dumas; who state, that they are to be found in the semen of all animals capable of propagation, but that they are absent in that of the mule, and that of other animals who may be sterile from age or season of the year. Very slight differences are noticed in the seminal animalcules of different species; but they all agree in having slightly oblong and flattened heads, with lengthened tails, tapering so as to become nearly or quite invisible with the best glasses: they possess active powers of motion, and are evidently endowed with sensation. No trace of organization has yet been discovered in them, probably on account of their extreme minuteness. Whether essential to generation or not, they may be regarded as the parasites of the tubuli seminiferi. They are called *circoria seminis,* by Richard Owen, who classed them with the trichina." 213.

Many feigned complaints have been referred to parasitical worms, and it is necessary that medical men should become acquainted with their natural history and character. That must be done by a reference to the works of elminthologists. We have not, therefore, entered into those characters, or that history here, as their description would be prolix if complete, and almost useless if partial. We have given a sort of resumé of the parasitical worms of man, and we must refer the curious reader for more circumstantial information to the proper sources.

Of the *Parasitic Insects* it is requisite to speak. The most prominent are the various species of pediculi, or lice—the pediculus corporis—pediculis capitis—and pediculus pubis;—the pulex irritans, or flea; the cimex lectuarius, or bug; the pulex penetrans, an American insect, which makes its way under the skin of the hands and feet, deposits its eggs there, and produces troublesome tumors; several species of acarus, as the acarus scabiei, or itch insect.

Dr. Hodgkin concludes the lecture by an examination of the obscure, indeed mysterious mode, in which parasitical animals are developed in the tissues where they are found.

That some spring from ova accidentally or designedly introduced into the body of the animal they infest, cannot be doubted. The œstrus equi, for example, deposits her eggs about the knees and shoulders of the horse, where they may be within reach of his tongue. The beast is thus the means of introducing the parasite into his own system.

But it is not so clear how the entozoa are generated in the tissues where they are found. Where proof is wanting, fancy is active, and conjecture does not hesitate to advance her claims. It has been supposed, *that* the entozoa may exist in other situations, under different appearances, their forms being changed when they become parasitic—*that* they are the result of equivocal or spontaneous generation—*that* some of the classes of parasitic animals, for instance the hydatid, are really not possessed of a separate vitality at all—*that* they *are* animals propagated by germs of invisible minuteness, which pervade and are received with our air and food.

It must be confessed, that there is something to be said for, and much against each of these hypotheses. The present state of our information on the subject does not warrant a positive opinion any way, and we dismiss an inquiry which can lead to no satisfactory results.

We turn to Dr. Carswell. The fasciculus of his work which lies before us, is occupied with the consideration of the:—

### III. Analogous Tissues.

Such is the name given to all solid morbid products, which resemble the natural elementary tissues of the body. Including bloodvessel under the name of vascular tissue, they are the following:—Vascular, including erectile—cellular, including fat—serous—mucous—cutaneous—cuticular, including hair and nails—fibrous—fibro-cartilaginous—cartilaginous—osseous.

1. *Origin of Analogous Tissues.*—The most frequent source of them is the spontaneously coagulable part of the blood, or the fibrine. To the tissues which have their origin in this, the terms *analogous, accidental, adventitious,* or *pseudo-formations* is applied. Another and a different source of these tissues, is the conversion of the primary or existing elementary tissues into them. Thus cartilage is converted into bone, the cellular into serous or fibrous tissue, and so on. The tissues so resulting, are called *analogous transformations.*

Analogous formations and transformations admit each of a sub-division. The analogous formations may originate either in the coagulable part of the blood, independently of inflammation; or in the coagulable lymph which is a product of that state. The analogous transformations may be the consequence of the conversion of a tissue of a lower into one of a higher grade, or the reverse. The change of cellular into serous tissue offers an example of the first sort of transformation, which is designated the *ascending* series—that of osseous tissue into cartilaginous, fibrous, and cellular in succession is an illustration of the opposite, or *descending* series.

A. *Analogous Formations originating in the Fibrine of the Blood.*—The evidence of this is chiefly drawn from the effects which follow the arrest of the blood in the heart or bloodvessels, or, its effusion into the substance of an organ. We will suppose the blood so arrested in a vessel. The changes which ensue are well described by Dr. Carswell. The first change which the blood is observed to undergo in these circumstances is coagulation, the extent of which, in an artery, is almost always determined by the situation of the first branch of considerable size, sent off from the obstructed vessel, between the ligature and the heart, but which, in the veins, varies with the situation of the obstacle, and the greater or less facilities afforded for the development of a collateral circulation. Whatever may be the extent of the coagulation, the subsequent changes which take place in the blood are as follow: The coagulum acquires gradually an increase of density, which is accompanied by the removal of the red colouring matter of the blood. The fibrine becomes thus more and more apparent, and is recognised by its pale straw-colour, and more especially by the manifestation of its plastic properties, whereby it assumes, almost from the commencement, a laminated or fibriform arrangement. In this, the early stage of what may be called the process of organization of the fibrine, there is one circumstance which is peculiarly interesting, not only because it enables us to explain the origin and mode of formation of some analogous tissues, but because it shews that the vital endowments or plastic properties of fibrine, under the circumstances in which we are now considering it, are of the same kind as those of coagulable lymph, however much they may vary in degree. The circumstance is question in the tendency of the fibrine to accumulate at the circumference of the coagulum.

The process of organization of the fibrine may go on throughout the whole of the coagulum, or it may stop at some distance from the central portion of it. In the first case, the vessel becomes permanently obliterated, for the fibrine, as it acquires a more perfect organization, unites with the internal surface of the vessel, contracts gradually, and converts it into a fibrous cord. In the second case, the vessel may remain pervious to a limited extent, for the unorganized central portion of the coagulum, or the serum and red colouring matter, or, as sometimes happens, a grumous milky-looking fluid, found in this situation, may be slowly impelled onwards by the same contractile property of the eccentric tissue which produced the cord-like obliteration of the vessel in the former case, and thus the admission and circulation of the blood be re-established.

The vascular organization of the fibrine is another phenomenon of both interest and importance. It takes place under two circumstances:—first, when the circulation has been arrested by a mechanical cause which has no tendency to induce inflammation in the obstructed vessel, and the only appreciable organizable product is the fibrine of the coagulum; secondly, when the circulation has been arrested by a ligature, or by inflammation of the vessel, and the organizable product occurs not only in the form of fibrine, but also in that of coagulable or plastic lymph. In neither case has Dr. Carswell been able to discover the primary and independent formation of bloodvessels *in* this substance. On the contrary he has always found the vessels which it contained existing in the form of capillaries, derived from the collateral branches of the obstructed or obliterated vessel; for they were not only continuous with the latter, but, penetrating the fibrine at the circumference of the vessel at various points by a single branch, afterwards assumed a capillary distribution in the laminated and fibriform structure of this substance. Although the presence of plastic lymph is highly favourable to the vascular organization of the fibrine, yet Dr. Carswell cites several reasons for concluding that it is not absolutely necessary for it. He conceives that the fibrine, acting as a foreign body, induces some modification of the capillary circulation of the walls of the vessel.

Dr. Carswell proceeds to describe the formation of polypi of the heart and arteries and of phlebolites, illustrative as it is of the preceding doctrine. On the former head we perceive nothing to detain us. But, as Dr. Carswell remarks, the formation of what are denominated *phlebolites*, is a remarkable example of the gradual conversion of the fibrine of the blood in the veins, into isolated round or ovoid bodies of a stony hardness. These bodies vary from the size of hemp-seed to that of a pea; they consist at first of a clot of blood, which, as it loses its red colour, assumes all the physical characters of fibrine. This is disposed in several concentric layers, which acquire considerable density, approaching occasionally to that of fibrous tissue, without, however, presenting its other physical characters, and always terminating in a degree of hardness equal to that of stone. In this state, as well as at their commencement when in the form of a fibrinous coagulum, they are contained in a serous envelope, and lie merely in juxta-position with the lining membrane of the vein. They are generally found in the broad ligament of the uterus.

"I shall only further illustrate this interesting part of our subject by a passing notice of those formations which arise in the fibrine of the blood in its course through the arteries, apparently under the influence of a physical agent; but of what kind, or the manner in which it operates, is not known. Thus, needles which have been made to transfix an artery, become as it were encrusted with fibrine; spiculæ of bone projecting into the cavity of an artery, have sometimes appended to them pediculated pyriform masses of fibrine, an inch in length, half an inch in breadth, and an eighth of an inch in thickness. They present a fine radiated structure, and lie in the direction of the vessel with their basis from the heart, having received this direction from the current of the blood during their formation. An extremely interesting example of this kind is represented in Fasciculus VII. Plate III. *fig.* 2. It is on the same principle that a coagulum forms around a mesh of fine thread when introduced into the cavity of an artery, as in the case represented in Plate III. *fig.* 3. The experiment was performed on a dog, in the following manner, by M. Magendie:—After laying bare a portion of the axillary artery, a needle, armed with several fine threads, was introduced obliquely through the walls into the cavity of the vessel. The point being carried upwards, it was withdrawn in that direction, and the thread pulled till its extremity dropped within the vessel, when it was left floating in the stream of blood. On the following day the circulation through that part of the vessel had ceased, and on the third day the animal was killed. Externally, that portion of the vessel submitted to the experiment was red, swollen, and felt firm, and when laid open, contained a fusiform coagulum, about an inch and a half in length, suspended near its middle by the thread, enclosed within it, and passing through its centre in the direction of the circulation. The lining membrane of the artery was not inflamed, nor was there the slightest trace of plastic lymph."

B. *Analogous Formations originating in the Plastic Lymph of the Blood.* The plastic lymph separated from the blood is found by analysis to be essentially composed of fibrine, and enjoys two very remarkable properties—that of spontaneous coagulation—and that of becoming organized, and subservient to the formation of more perfect products.

C. *Formation of Bloodvessels.* This naturally occupies the attention of our author. He gives a very good account of the phenomenon, and it richly merits all the attention it receives. This organization is one of the most beautiful, one of the most characteristic, apparently one of the most complex, yet, probably, when it shall be thoroughly understood, it will turn out to be one of the most simple, of the vital processes.

Dr. Carswell takes the lymph effused on the free surface of a serous membrane, as the best adapted for examination. The stages (we shall abridge and methodize them) are these:—

1. Besides the redness and vascularity which characterise inflammation of the pleura, and which appear at first in isolated spots situated in the sub-serous cellular tissue, but which, at a subsequent stage of the inflammation, extend to the serous membrane itself, the free surface of this membrane presents a villous or granular appearance, particularly at those points where the redness and vascularity are most marked. The pleura has now undergone important changes, especially at these points; it appears considerably increased in thickness, is so soft as to be easily removed by passing the edge of a scalpel over it, and contains small specks or striæ of blood. This softening of the pleura increases with the progress of the inflammation, and is probably, essential for the establishment of the vascular communication between the membrane and the lymph.

2. The plastic lymph which forms on the surface of the pleura appears first in the most inflamed points, but gradually increasing in quantity with the extension of the inflammation, forms a continuous layer of various thickness and extent. At first it is of the consistence of mucus or thin cream, of a pale yellowish-white or grey colour, and gradually acquires an increase of consistence amounting to that of coagulated albumen or pure fibrine. It is always most consistent the nearer it is examined to the surface of the pleura, the serum which it contains, when first effused, being expelled by its power of spontaneously coagulating, and therefore is most abundant in the more recent deposit, or is collected in the cavity of the pleura. Although presenting sometimes externally a cribriform or rather reticulated appearance, it appears to possess a fine cellular structure; for when it is not firmer than jelly, it may be suspended between the fingers without much of the serum with which it is then loaded making its escape; and when this fluid is forced out by compressing a quantity of the recent lymph in the hand, it resembles a mass of cobwebs moistened with water. The quantity of lymph organized or organizable varies with its vital power and with its distance from the inflamed membrane. The unorganized lymph may hang in shreds, or fall en masse to the dependent parts of cavities, where it is found for that reason in the greatest quantity.

3. Sometimes at a very early period, at others not until after several days, bloodvessels make their appearance in the plastic lymph of serous membranes. At a very early period of their formation, they are often so numerous as to give an almost uniform red colour to the false membrane in which they are distributed, and are always more numerous at this than at any subsequent period. They diminish in number and size as the false membrane acquires a more perfect organization, and scarcely a trace of them sometimes remains when it has assumed the structure of cellular and serous tissue. It is only in the false membranes extending from one serous surface to another that Dr. Carswell has been able to determine the disposition of the vessels. They are then distinctly seen to consist, each of a flexuous or tortuous trunk, the opposite extremities of which divide and subdivide into numerous minute branches, like the hepatic and abdominal divisions of the vena portæ, which communicate with, and can be easily injected from, the general vascular system.

4. We need not say that the precise mode in which bloodvessels are developed in lymph, has excited much attention, and some difference of opinion. The latter cannot be wondered at. The subject of examination is exceedingly minute, and exact observation is most difficult at that period when it is most necessary, the very commencement of the process.

Two leading theories divide physiologists and pathologists. The first maintains that the new vessels are prolongations of the old. The second contends that the new vessels are originally formed in the lymph, and that they afterwards communicate with the capillaries of the general circulation. Each hypothesis has its supporters and its facts, and it is difficult in the existing state of information to determine which is the true one. Certainly, that which supposes the extension of the old vessels to be the main agent in the production of the new, is most consistent with mere reasoning and with probability. But be this as it may, the minute investigations of Gruithausen, Daelinger, and Rattenbrunner on the changes actually observed, are not unworthy of attention. They are thus described by Dr. Carswell :—

"The first trace of the new bloodvessel appears in the form of a spot, which soon assumes a more defined and circumscribed shape. In this, granules make their appearance, which present an obscure motion, and the canal in which they are contained becomes elongated and assumes a crescentic form, the cornua of which are turned towards the extremities of the old vessels. The motion of the granules becomes more distinct as the channel in which they move increases in length. The cornua at last reach the extremities of the old vessels, whence the blood is transmitted through the former, and a vascular connection thus established by a kind of loop, between a neighbouring artery and vein. It is in this manner that a connection is established between the new vessels formed in the plastic lymph on the surface of a wound, and the vessels of the general circulation on the same side, but it does not appear to have been ascertained in what manner it is accomplished between the new vessels of both sides, after complete union of the wound has taken place. A similar mode of formation of new vessels has been observed to take place in the plastic lymph which has been effused into the parenchyma of organs, and on the surface of sores during the process of granulation. In the latter situation the lymph deposited on the surface of the sore presents numerous stellated red points, which undergo changes similar to those observed in the lymph of inflamed serous membranes, that is to say, they appear to give rise to the formation of new vessels, which afterwards become connected with the original vessels. The former are prolonged, at isolated points, in the lymph, and produce the vascular projections called granulations. The structure of these was first demonstrated by Professor Thomson of Edinburgh, who showed, by means of injections, that the vessels which they contain, proceeding from the vascular basis of the sore, arrive at their extremities, and there unite in the form of loops."

We now proceed to the consideration of the analogous tissues themselves. The first alluded to by Dr. Carswell is :—

II. *Erectile Tissue.* This is common on the surface of the body, forming nævi, aneurysm by anastomosis, &c. It may, like the natural erectile tissue, consist of a spongy or cellular structure, intercepted by fibrous tissue ; or, which is more frequent, it may present an almost inextricable network of arteries and veins, sometimes the one, sometimes the other of these sets of vessels predominating. The only viscus in which Dr. C. has seen this tissue developed has been the liver. In that it assumes the form of a round tumor, varying from the size of a large pea to that of a small orange. Dr. Carswell has never seen it form after birth except at the lips and at the margin of the anus. There cannot, however, be a doubt that erectile tissue is not very unfrequently developed on the face and scalp, at a considerable period after birth.

III. *Cellular Tissue*. This is constantly developed. Between serous membranes it is familiarly known under the name of adhesions. We need not dwell on *them*. Our author draws attention to the important part which such adhesions play, when they occur between the uterus, fallopian tubes, ovaries, and surrounding parts. They are a not unfrequent and a very obvious cause of sterility. They produce, according to their situation and mode of attachment, either anteversion or retroversion of the uterus; they fix the Fallopian tubes in situations in which the fimbriated extremities cannot reach the ovaries; or they envelop the fimbriated extremities in such a manner as to render them quite impervious, (which is always the cause of dropsy of these tubes); or, lastly, they cover the ovaries so completely that impregnation is rendered impossible.

Dr. Carswell directs attention to an error not unlikely to be committed.

"The accidental cellular tissue, whenever it presents a free serface, is always covered by a serous membrane of new formation; and hence arises a frequent source of error regarding the seat of some morbid products. When these products are contained in the accidental cellular tissue, and, consequently, are covered by the new serous membrane, they are often described as being situated on the outside of the original serous membrane, as, for example, in the subpleural or subperitoneal cellular tissue; and this error is the more likely to be committed when the original serous membrane has nearly or altogether disappeared in the situation of cellular adhesions. This is often observed in extensive cellular adhesions of the pleura, peritoneum, and pericardium; and so complete is this change in some cases of chronic pericarditis, that hardly a trace even of the fibrous layer of the pericardium is left, a circumstance which, as Lobstein observes, has given rise to erroneous statements regarding the absence of this sac."

S. V. der Kolk is said to have injected the absorbents of accidental cellular tissue with mercury.

On the *adipose tissue* there is little said. But we must pause at—

IV. *Serous Tissue*. Under this head, we are naturally presented with an account of those cysts which are formed of adventitious serous membrane. These must, of course, be distinguished from the cysts which are enlargements of what naturally exist, as of the Graafian vesicles, of bursæ, of ganglia, or of obstructed ducts or follicles.

"The adventitious serous cyst, properly so called, consists of a serous membrane, having the form of a shut sac, the external surface of which has a cellulo-vascular connexion with the contiguous tissues, the internal or free surface being in contact with the substances which it encloses. This connexion may be formed between it and the natural cellular tissue of organs; or between accidental cellular, fibrous, cartilaginous, and osseous tissues, all of which not rarely contribute, particularly in the liver, to form the entire walls of the cyst. Or, lastly, it may be connected with the heterologous formations, especially the carcinomatous, when they exist as pediculated tumours, and then constitutes their capsular covering, in the form of a reflected membrane, extending over their whole surface to the point of their attachment. It is from this circumstance, I believe, that such tumours have been described not only as being contained within, but as being produced by, this kind of serous-cyst, the anatomical and physical characters of which have been minutely described by Dr. Hodgkin, and to which he attributes peculiar properties, the exercise of which, he believes, gives origin to many or most of the heterologous formations."

Dr. Carswell thinks, there are good grounds for believing that the origin of the adventitious serous cysts is inflammatory, or dependent on causes which excite inflammation of the cellular tissue of circumscribed portions of organs. The irritation of a bullet lodged in a organ, tuberculous matter, the presence of entozoa, may give rise to the effusion of lymph, and that becoming organized, will form a cyst; but, however formed, they are afterwards variously affected by disease, and may contain a great variety of substances. They may envelope, for instance, the heterologous or malignant formations—serous, albuminous, mucous, or puriform fluids—lymph, fibrine, or blood variously modified—tuberculous matter, cholesterine, and some other anomalous products. The ovaries, testes, and mammæ are more frequently the seat of them than any other organs are. They are frequent in the kidneys of old persons; but very seldom have they been seen in the brain or lungs. In the organs of reproduction they are generally collected together in clusters; in the brain and lungs solitary; and in the kidneys in both forms, but more often solitary.

V. *Mucous Tissue.*—The accidental formation of mucous tissue is observed, 1st, in situations in which the natural mucous membrane does not exist, as in those solutions of continuity which follow inflammation, ulceration, or mortification of the cellular tissue, and which afterwards become the seat of chronic abscess, whether in the form of a shut cavity, or communicating with the external surface of the body by what is called a *fistulous passage;* 2d, in the situation of a natural mucous membrane which has been destroyed, the cicatrix which supplies its place consisting of a mucous tissue.

The mucous tissue in either case originates in the lymph, deposited in the inflamed cellular tissue, and subsequently organized. The following is the description of the change, presented by our author.

"In the first stage of the process, the cellular tissue is consolidated by the lymph, of which there is also a layer on its denuded surface; it then acquires a vascular structure, which often produces a deep red colour, accompanied by an irregular granular appearance in this situation, and here also it afterwards gradually assumes either a uniform, smooth, glossy, or rough villous aspect, or that of simple mucous membrane. The mucous tissue thus formed adheres intimately to, or is confounded with, the cellular tissue, which has either regained its natural state or has acquired a considerable degree of density. The colour which it presents varies much in abscesses and fistulæ, according to the degree of inflammation, the quantity of blood which it contains, and the chemical action of the gaseous products to which this fluid is often subjected. It is generally, however, of a reddish or greyish blue colour in these situations, and in the intestines differs so little from that of the natural mucous membrane, that the cicatrix which it forms would escape detection if not carefully sought for."

In no situation, does the accidental mucous tissue present any trace of muciparous follicles or glands, while its villi differ both in form and structure from those of natural mucous membrane. It both secretes and absorbs, the secretion sometimes resembling the natural mucus. Villermé has found the accidental mucous membrane of fistulæ lined with an epithelium continuous with the epidermis. The accidental, like the natural mucous tissue, is not disposed to adhesion when inflamed, its secretion not being organizable. To produce adhesion we must destroy it. Hence many of the operations for fistulæ and chronic abscesses.

VI. *Cutaneous Tissue.* The process of cicatrization has been too frequently described to permit us to expect any thing on this head. We may merely hint that, although the papillæ are sometimes partially developed in the new skin, the muciparous glands and the hair do not seem to be reproduced, if the destruction of the cutis has been complete.

" *Hair.* The accidental development of hair is seen to take place most frequently on those parts of the body affected with nævi, where it is often very abundant, fine or coarse, short or long, and of various colours. But here, like the nævi which it accompanies, it is to be considered rather as a congenital than an accidental formation. So also is the hair so often found in ovarian cysts. That found in subcutaneous cysts, as those of the scalp and eyelids, is probably the result of accidental development, as it arises from the interior of these cysts, which is lined by the reflected cutaneous tissue. The most remarkable examples of the accidental development of hair are met with in some portions of the natural mucous membrane, as that covering the caruncula lachrymalis, the tongue, and urinary bladder. In the latter situation, however, it has always been found loose, without bulbs, seldom more than an inch in length, and has generally been passed in the urine with the phosphate of lime, sometimes in great quantity."

*Nails.* These are reproduced, as they were formed, by the cutis which secretes them. But new nails are often deformed. A "horny tissue," sometimes grows from cicatrices from the stump, for example, of an amputated finger. This is concrete albumen, the secretion of the inflamed cutis. Horny productions may, it is well known, grow from the skin, and particularly from the scalp. They appear to be altered and consolidated sebaceous secretion.

VII. *Fibrous, Fibro-cartilaginous, and Osseous Tissues.* These occur under nearly similar circumstances, and follow each other nearly in the order set down. This agrees with what is observed in the natural production of bone. The following account of the reparation of bone, though long as an extract, is short as a description, and in the latter sense is so true as well as brief, that we cannot resist the opportunity of presenting it unbroken to our readers.

" Whether the formation of new bone is to effect the reunion of a fracture, or the restoration of a loss of substance of the old bone, the process by means of which it is accomplished is essentially the same as that which takes place in lesions of a similar kind of the soft parts. Inflammation, the effusion of plastic lymph, the celluло-vascular organization of this, and its gradual conversion into fibrous, fibro-cartilaginous, cartilaginous, and osseous tissues, follow each other in succession. The material out of which the new bone is formed is the plastic lymph, which is furnished by the periosteum, medullary membrane, surrounding cellular tissue, and the substance of the bone itself, according as the inflammation by which it is preceded affects one or all of these parts, a circumstance which will depend chiefly on the original seat or extent of the injury or disease. The parts which contribute most to the formation of the new bone, and in which this process is first established, are those whose anatomical and physiological conditions render them most susceptible of inflammation, or in other words, those whose vital endowments are greatest, and which, consequently, furnish the most abundant supply of plastic lymph. These are, 1st, the periosteum; 2d, the surrounding cellular tissue; 3d, the medullary membrane; 4th, the substance of the bone. It is not until some time after the three former have been the seat of inflammation, and the plastic lymph effused by them has become organized, that the substance of the bone participates in the same change, and takes a part in the regenerativ eprocess. The plastic lymph is always furnished in greatest abundance by the periosteum and surrounding cellular tissue, as is clearly shown by the appearances which accompany fracture of a long bone. It is found much more abundant around the circumference, and in the situation of the fracture, than elsewhere; and if the inflammation has been severe, forms a mass of considerable extent projecting outwards, and dipping between the fractured extremities, where it meets with that effused by the medullary membrane. Hence, also, the cellulo-vascular organization of the lymph, and the subsequent changes which it undergoes, are effected in the same relative order, the formation of the new tissues being far advanced or completed around the circumference of the bone, before it has commenced or has made much progress between the fractured extremities, and especially between these and the uniting medium. It is on account of the ossific process being first completed in the tissues around the bone, that these are said to form the *provisory callus*, a provision, indeed, which affords time for the development of a vascular connexion between them and the extremities of the bone, and the accomplishment of a permanent bony union. 16.

Dr. Carswell very justly observes that an arrest of the reparative process may take place, and the fragments of bone may be united only by fibrous or fibro-cartilaginous substance. He might have added that the sort of cyst in which the fractured ends are occasionally enlarged, as in a capsule, while those ends themselves become rounded, as in an articulation, is nothing more than an example of the same phenomenon, an arrest of the reproductive action.

Dr. Carswell gives a sketch of the changes which take place in necrosis. There is nothing in it to detain us.

Prior to quitting the analogous formations, Dr. Carswell makes a few observations on the tendency to contraction displayed by the fibrous tissue, and on the occurrence of the cartilaginous tissue in the form of isolated round or ovoid bodies in the cavities of serous or synovial membranes.

A. The fibrous tissue, says Dr. Carswell, whether originating in the plastic lymph of serous membranes or of that of the cellular tissue of organs, and when it does not undergo any further change towards the formation of the fibro-cartilaginous or osseous tissue, has a greater or less tendency to contract upon itself, and consequently to modify the nutrition, bulk, form, and situation of the parts with which it is connected. Atrophy of a lung, when this organ is covered by a fibrous envelope in chronic pleurisy; stricture of the intestines after cicatrisation of ulcers which had destroyed the muscular coat around the circumference of the tube; displacement of the fingers, of the lower lip and jaw, &c. after burns—are effects of the contractile property of this tissue. Every body knows the mischief occasioned by the contraction of the cicatrices of burns. Dr. Carswell has seen it produce complete closure of the mouth and nostrils, and death from inanition.

"When treating of the vascular organization of lymph in the form of membranous adhesions and granulations, I stated that these diminish in bulk, and acquire an increase of density in proportion as their vascular element becomes less apparent and they assume the fibrous character. These changes I regard as the early indices of the contractile property which afterwards becomes so apparent in this tissue in the cicatrices of burns, and indeed of most cicatrices which succeed to a considerable loss of substance, more especially when the process of regeneration is accomplished by granulation. And it is also to be observed, that the more exuberant and vascular the granulations, the more certainly is the contractile property acquired, and its effects manifested in the cicatrix which follows. These conditions of the granulations are decidedly much more conspicuous after burns than after any other lesion, and it is on this account, as well as from the facts already noticed, that I feel disposed to ascribe the existence of this property to an excess of the vascular in combination with the fibrous tissue. This hypothesis derives support from the fact that the contractile property of this tissue is slight in degree when the process of granulation is retarded, or its activity repressed, before cicatrization is allowed to take place."

This hypothesis is ingenious. Whoever has watched carefully the process of cicatrisation after burns, and the changes of the cicatrix itself, must have observed that the activity of the vessels does not cease immediately after the new skin is formed. It is not uncommon for deposition to go on under the newly-formed cuticle, and a cicatrix which at first was not raised above the level of the surrounding surface, becomes in a short time elevated and welted, independently of any appreciable contraction. Cicatrices, too, are not unfrequently the seat of chronic inflammation. These circumstances go to prove that the cicatrix is in the first instance the seat of a good deal of vascular action, and it is highly probable that Dr. Carswell's conjecture is correct, and that the subsequent contraction is very much in the ratio of that action.

B. The "loose cartilages" of joints are ascribed by Dr. Carswell to lymph effused from the synovial membrane, organized in connexion with that membrane, converted into the cartilaginous tissue, and finally detached. John Hunter ascribed them to coagulum thus organized. Dr. Carswell has seen no fact corroborating Hunter's supposition. But he has found fibrous tumors in the cavity of the abdomen, which had been originally situated beneath the peritoneum covering the fundus and broad ligaments of the uterus, and he once saw a small, pyriform, fibrinous coagulum, covered by a prolongation of the peritoneum, and supplied with several bloodvessels from this membrane, in a rabbit (a case similar to that observed by John Hunter,) which, at some future stage of its development, would no doubt have become detached and fallen into the cavity of the abdomen.

It is possible, he adds, that the loose cartilages may also originate in the manner described by Béclard, that is to say, the cartilaginous formation having taken place in the cellular tissue, behind the synovial membrane, carries before it, in proportion as it increases in bulk, a portion of this membrane, which, having become pediculated, is at last detached, and drops into the cavity of the joint.

VIII. *Analogous Transformations.* The analogous formation is the addition of one tissue to another. The analogous transformation is the conversion of one tissue into another. The transformations are subjected to the same laws which preside over the regular development of the natural tissues, and observe the same order or gradation by which these pass the one into the other at different periods of fœtal life and in different animals. Thus the cellular tissue is transformed into serous or fibrous tissue, and this into fibro-cartilaginous, cartilaginous, and osseous tissues in succession, constituting, as already observed, the ascending series; and when taking place in the reverse order, the descending series of this class of analogous tissues. Hence it follows that those tissues which, in the physiological condition, do not observe this order of succession, are not found to do so in the pathological condition. Thus bone, muscle, nervous matter, &c. are not successive developments of each other in the physiological condition, and do not occur in the pathological.

All the transformations of the ascending series may originate in the cellular tissue, in which and in the fibrous they are most frequent. All those of the descending series may originate in other tissues and in glandular and parenchymatous organs, where they are most rife. These organs, indeed, are susceptible of undergoing only the second series of transformations, as well as the muscular and nervous tissues. The mucous and cutaneous tissues are subject to both, being convertible the one into the other, as also the cartilaginous and osseous, the fibrous and cellular tissues.

Our author offers a description of the fibrous tumors of the uterus on which, however, it is unnecessary for us to dwell. He has never seen these tumors degenerate into malignant disease.

The osseous transformation, which in Dr. Carswell's opinion, is exclusively confined to the cellular, fibrous, fibro-cartilaginous, and cartilaginous tissues, usually makes its appearance after the age of forty in the cellular and fibrous tissues of the vascular system. No probable reason, as he observes, has been assigned for its occurring so frequently in the arteries and so seldom in the veins, except perhaps that the former, from the greatly increased exercise of their function, compared with that of the latter, arrive sooner at that condition which constitutes the period of decay, a condition which in this as well as in other tissues is so favourable to its production. The osseous transformation occurs most frequently in the aorta, the arteries of the brain, and lower extremities; much more seldom in the valves of the right than in those of the left side of the heart; rarely in the pulmonary artery and veins. Its seat in the arteries is the cellular tissue which separates the inner membrane from the middle coat, and in the middle coat itself. It appears in the form of small circular, oval, or irregular patches, of a dull white or yellowish colour, of the consistence of cartilage, in the former; and in that of plates, spiculæ, or rings in the latter, occupying a greater or less extent of the vessel, portions of which are sometimes converted into an osteo-cretaceous tube resembling the trachea of a bird. Over the deposit the inner tunic of the vessel is ruptured or ulcerates, and aneurysm or hæmorrhage is well known to be the consequence.

"The osseous transformation is always very imperfect in the vascular system. It contains a much greater quantity of earthy matter than the natural bony tissue, is therefore much harder, and in general dry and brittle. It rarely presents, in any situation in which it is formed, either medullary cavities or periosteum. Cartilage and fibro-cartilage are perhaps the only tissues in which this occasionally occurs."

The fatty transformation of organs is the last subject which occupies our author. He believes that such a transformation does really take place in some organs, as the muscles and liver, and sometimes in the kidneys and pancreas.

"It is most marked in the muscles of the inferior extremities of old persons affected with paralysis, and which have long remained in a state of absolute rest. In such cases I have found the affected muscles but little altered, except in their colour and chemical composition. They are of a pale-white or straw-colour, diminished rather than increased in bulk, and the arrangement of their fibres perfectly distinct. Indeed, the larger bundles of the muscular fibres, and their subdivision into smaller ones, can easily be traced with the scalpel. When dissecting the muscular fibres, or when these are pressed between the fingers, a clear oily fluid oozes out, and which, when a portion of the muscle has remained some time in alcohol, collects in considerable quantity on the surface of this spirit. According to Lobstein, the chemical analysis of muscular tissue which has undergone the fatty degeneration affords an oily matter, a substance resembling boiled muscle, gelatine, adipocire, and solid fat."

This concludes our account of the present fasciculus. The plates are illustrative of the text and excellent in point of execution. Dr. Carswell deserves encouragement, and we trust that, despite of the apathy of the Profession on matters which are looked on as more abstractedly scientific than immediately practical, he will obtain it.

In our next Number we will return to the inexhaustible subject of pathological anatomy and select some series of changes for description.

